# N-Myristoylation by NMT1 Is POTEE-Dependent to Stimulate Liver Tumorigenesis *via* Differentially Regulating Ubiquitination of Targets

**DOI:** 10.3389/fonc.2021.681366

**Published:** 2021-05-31

**Authors:** Guoqing Zhu, Feng Wang, Haojie Li, Xiao Zhang, Qi Wu, Ya Liu, Mingping Qian, Susu Guo, Yueyue Yang, Xiangfei Xue, Fenyong Sun, Yongxia Qiao, Qiuhui Pan

**Affiliations:** ^1^ Department of Clinical Laboratory Medicine, Shanghai Children’s Medical Center, School of Medicine, Shanghai Jiaotong University, Shanghai, China; ^2^ Department of Gastroenterology, Shanghai Tenth People’s Hospital of Tongji University, Shanghai, China; ^3^ Department of Clinical Laboratory, Shanghai Tenth People’s Hospital of Tongji University, Shanghai, China; ^4^ Shanghai Institute of Thoracic Oncology, Shanghai Chest Hospital, Shanghai Jiao Tong University, Shanghai, China; ^5^ Department of General Surgery, Shanghai Tenth People’s Hospital of Tongji University, Shanghai, China; ^6^ School of Public Health, Shanghai Jiaotong University School of Medicine, Shanghai, China

**Keywords:** post-translational modifications, N-myristoyltransferase, ubiquitin E3 ligase HIST1H4H, proteomics, liver cancer

## Abstract

**Background:**

A tremendous amount of studies have suggested that post-translational modifications (PTMs) play pivotal roles during tumorigenesis. Compared to other PTMs, lipid modification is less studied. Recently, N-myristoylation, one type of lipid modification, has been paid attention to the field of cancer. However, whether and how N-myristoylation exerts its roles in liver tumorigenesis still remains unclear.

**Methods:**

Parallel reaction monitoring (PRM) was conducted to evaluate the expression of protein modification enzymes in paired tissues. Liver conditionally knocking NMT1 out mice model was used to assess the critical roles of N-myristoylation during liver tumorigenesis. Proteomics isobaric tags for relative and absolute quantification (iTraq) was performed to identify proteins that changed while NMT1 was knocked down. The click chemistry assay was used to evaluate the N-myristoylation levels of proteins.

**Results:**

Here, N-myristolyation and its enzyme NMT1, but not NMT2, were found to be critical in liver cancer. Two categories of proteins, i.e., N-myristolyation down-regulated proteins (NDP, including LXN, RPL29, and FAU) and N-myristolyation up-regulated proteins (NUP, including AHSG, ALB, and TF), were revealed negatively and positively regulated by NMT1, respectively. Both NDP and NUP could be N-myristolyated by NMT1 indispensable of POTEE. However, N-myristolyation decreased and increased stability of NDP and NUP, respectively. Mechanistically, NDP-specific binding protein RPL7A facilitated HIST1H4H, which has ubiquitin E3 ligase function, to ubiquitinate NDP. By contrast, NUP-specific binding protein HBB prevented NUP from ubiquitination by HIST1H4H. Notably, function of RPL7A and HBB was all NMT1-dependent. Moreover, NDP suppressed while NUP stimulated transformative phenotypes. Clinically, higher levels of NMT1 and NUP with lower levels of NDP had worse prognostic outcome.

**Conclusion:**

Collectively, N-myristolyation by NMT1 suppresses anti-tumorigenic NDP, whereas it stimulates pro-tumorigenic NUP by interfering their ubiquitination to finally result in a pro-tumorigenic outcome in liver cancer. Targeting N-myristolyation and NMT1 might be helpful to treat liver cancer.

## Background

The correct functions of the most mature proteins require post-translational modifications (PTMs), including, but not limited to, ubiquitination, phosphorylation, acetylation, and lipidation (1). A tremendous amount of studies have suggested that PTMs play pivotal roles during the regulation of various physiological and pathological processes such as protein stability maintenance and tumorigenesis (2, 3).

PTMs occur *via* being catalyzed by the relevant elements to covalently attach to the amino acid residues of the specific proteins (4). Other than phosphate, methyl, acetyl, and sugar, lipid attachment is also one of the most common PTMs in cancer cells (5); however, the study focusing on lipid modification is not as popular as others. According to the identities of the attached lipid, there are at least four broad types of lipid modifications: myristoylation, palmitoylation, prenylation, and glypiation. Notably, covalent attachment of myristate, a 14-carbon saturated fatty acid, to the N-terminal glycine residue of targeted proteins, which is named as N-myristoylation, has been paid attention to the field of cancer (6). N-myristoylation is important for the biological functions of proteins. For example, ARF6 was demonstrated to be N-myristoylated on lysine 3 and recruited on membranes during the GTPase cycle to control ERK phosphorylation (7). Moreover, the N-myristoylation deficiency of AMPK inhibited its activation and lead to synovial tissue inflammation (8). However, the direct identification of N-myristoylation substrates was still very limited (9). Emmanuelle Thinon et al. conducted a global quantification of N-myristoylation by identifying more than 100 N-myristoylated proteins in cervical cancer cell, which further emphasized the pivotal role N-myristoylation in human disease (9). However, the N-myristoylation substrates in liver cancer cells still remains largely unidentified (10).

N-myristoylation can be catalyzed by the N-myristoyltransferase (NMT), which belongs to the Gcn5-related N-acetyltransferases (GNAT) superfamily. Up to now, two NMT isoezymes, i.e., NMT1 and NMT2, have been identified in mammals (11). Altered NMT expression has been linked to many types of cancer, including colon, breast, and gallbladder cancer (12); however, it is unclear whether it is involved in liver cancer. Although a number of studies support NMT to be regarded as a potential target for tumor therapy, whether and how N-myristoylation and NMT affect liver tumorigenesis is still poorly understood.

Prostate, Ovary, Testes, and Embryo (POTE) gene is a newly detected gene family located on chromosome 21 (13). POTE Ankyrin domain family member E (POTEE) is a paralog and contains ankyrin and spectrin domains (14). Previous studies reported that POTEE was only weakly expressed in normal tissues, but its expression was significantly elevated in tumor cells (15). Anchor-mediated ligation of POTEE participated in numerous biological activities by mediating physiologically important interactions between proteins and their adaptors for signaling transduction (16). For instance, POTEE was demonstrated to provide a platform for mTOR and Rictor binding and lead to the activation of mTORC2 in macrophages (17). However, the role of POTEE during PTMs of HCC still remains unknown.

Here, we revealed that N-myristoylation and NMT1 positively regulates liver tumorigenesis *via* controlling differential expression of two categories of proteins, which oppositely control transformative phenotypes. We also identified that POTEE is essential for NMT1-mediated N-myristoylation of target proteins. Moreover, HIST1H4H has been uncovered to be closely associated with the final outcome of target proteins that can be N-myristoylated.

## Materials and Methods

### Cell Culture and Vectors

The liver cancer cell lines SMMC-7721, HepG2, Bel-7402, Bel-7404, Huh7, and SK-Hep1 and hepatocyte lines THLE-3 and HL-7702 were purchased from the Cell Bank of the Chinese Academy of Sciences (Shanghai, China). All the cells were cultured in DMEM (Hyclone, Logan, UT, USA) supplemented with 10% FBS (GIBCO, Carlsbad, CA, USA) and penicillin/streptomycin (GIBCO). Cells were treated with Cycloheximide (CHX, Sigma, St Louis, MO, USA) at a final concentration of 0.1mg/ml and MG132 (Cayman Chemical Co., Ann Arbor, MI) at a final concentration of 25 µM. NMT1- and NMT2-exprssing plasmids were purchased from Origene (Beijing, China). The plasmids expressing NMT1-sh1, NMT1-sh2, POTEE-sh1, POTEE-sh2, RPL7A-sh1, RPL7A-sh2, HBB-sh1, HBB-sh2, HIST1H4H-sh1, and HIST1H4H were purchased from Genechem (Shanghai, China). The expressing plasmids of LXN-HA, FAU-HA, RPL29-HA, LXN-Mut-HA, FAU-Mut-HA, RPL29-Mut-HA, AHSG-HA, ALB-HA, TF-HA, AHSG-Mut-HA, ALB-Mut-HA, TF-Mut-HA, POTEE-Myc, RPL7A-FLAG, HBB-FLAG, HIST1H4H-HA, and HIST1H4H-Mut-HA were purchased from Biolink (Shanghai, China).

### Mouse Experiments and Tissue Samples

The 8-week-old Balb/c male athymic nude mice were purchased from Bikai (Shanghai, China). For xenograft mouse experiments, Bel-7404 cells (5 × 10^6^ cells) under different treatments were subcutaneously injected into the athymic nude mice. The tumor volume was calculated as 0.5 × L × W^2^ (L indicating length and W indicating width). The tumor sizes were measured after mice were euthanized at 36 days after injection. For NMT1 liver-specific expressing, NMT1 knock-out mice were injected with NMT1-pLIVE plasmids [10 μg plasmids diluted in 2 ml *Trans*IT-EE Hydrodynamic Delivery Solution (MirusBio (Madison, WI, USA)] *via* tail within 5 s. As for the DEN/CCl4-induced liver cancer mice models, the mice were weighted and 100 µl/10 g DEN (4 mg/kg) was infused into the peritoneal cavity at the age of 14 days. After 6 weeks, 50 µl/10 g CCl4 (20%) solution was infused into the peritoneal cavity every 2 weeks, and the mice were euthanized 4 months later. For generation of NMT1 knock-out mice (NMT1^−/−^), mice carrying conditional knockout heterozygous for NMT1 (NMT1^flox/+^) were crossed with Albumin (*Alb*, specifically expressed in liver) *Cre* mice to generate the NMT1^flox/+^: Alb-*Cre* mice, which were then crossed with the NMT1^flox/+^ mice again to generate the NMT1^flox/flox^: Alb-*Cre* mice. Genotyping was performed *via* PCR using the following primers sequences: Forward (loxp): GCAGGTTAGACCCGACTCCC, and Reward (loxp): CCTAGCTTGCCACAGCTTGA; Forward (*Cre*): GGAGGATTGCTATAATTTTGAGG and Reward (*Cre*): GAACCACTTCTGTAATGCCTTTT. All the mouse experiments were performed according to the institutional guidelines of Shanghai Tenth People’s Hospital. Tumorous and adjacent normal liver tissues were acquired from the Shanghai Tenth People’s Hospital under institutional approval with informed written consent obtained from all patients.

### Immunohistochemistry (IHC), Western Blotting (WB), and Co-immunoprecipitation (co-IP)

IHC, WB, and co-IP were performed as described previously (18). The TMA slides were purchased from U.S. Biomax through the agent Alenabio (Xi’an, China). Briefly, IHC was performed using the Vectastain ABC kit (Vector Labs, Burlingame, CA, USA), and the primary antibodies used for IHC were: anti-NMT1 (Abcam, Hong Kong, China, #ab186123), anti-NMT2 (Abcam, #ab224045), anti-AHSG (Arigo, Hsinchu City, Taiwan, China, #ARG55425), and anti-RPL29 (Abcam, #ab67196). The protocol of WB was the conventional one, and is available elsewhere. The primary antibodies used for WB were: anti-NMT1 (Abcam, #ab186123 or Abcam, #ab70412), anti-NMT2 (Abcam, #ab224045), anti-TAMRA (Abcam, #ab171120 or Invitrogen, Carlsbad, CA, USA, #A6397), anti-ALB (Arigo, #ARG54036), anti-MTPN (Abcam, #ab90918), anti-GON7 (Abcam, #ab151146), anti-TF (Abcam, #ab82411), anti-AHSG (Arigo, #ARG55425), anti-GAPDH [Cell signaling technology (CST), #5174], anti-HA (CST, #3724 or #2367), anti-Myc (CST, #2276 or #2278), anti-FLAG (CST, #8146 or #2368), anti-LXN (Abcam, #ab154744), anti-RPL29 (Abcam, #ab67196), anti-FAU (Abcam, #ab63065), anti-HBB (Abcam, #ab214049 or #ab172019), anti-POTEE (Abcam, #ab108190), anti-RPL7A (Abcam, #ab155147 or Invitrogen, #MA5-27534), anti-Ub (CST, #3936 or #3933), or anti-HIST1H4H (CST, #8647 or #2935). The secondary antibodies used for WB were: anti-Rabbit IgG (HRP-linked) (CST, #7074) and anti-Mouse IgG (HRP-linked) (CST, #7076). The data of WB were recorded on X-ray film. As for Co-IP, cell lysates were incubated with protein A/G-Sepharose (Novex, Oslo, Norway) in Western/IP lysis buffer (Beyotime, Haimen, China). After preclearing for 1 h, the supernatants were incubated at 4°C overnight with the indicated antibodies. Immunoprecipitates were washed by lysis buffer at least five times, resuspended in SDS loading buffer (Beyotime), and boiled for 10 min before subjecting to WB analysis. The antibodies used for IP were: anti-FLAG (CST, #8146 or #2368), anti-HA (CST, CST, #3724 or #2367), anti-ALB (Arigo, #ARG54036), anti-TF (Abcam, #ab82411), anti-AHSG (Arigo, #ARG55425), anti-LXN (Abcam, #ab154744), anti-RPL29 (Abcam, #ab67196), anti-FAU (Abcam, #ab63065), anti-HIST1H4H (CST, #8647 or #2935), anti-HBB (Abcam, #ab214049 or #ab172019), or anti-RPL7A (Abcam, #ab155147 or Invitrogen, #MA5-27534).

### Cell Proliferation, Caspase3/7 Activity, Colony Formation Assay, and Quantitative RT-PCR

Cell proliferation, caspase 3/7 activity, colony formation capacity, and quantitative RT-PCR (qPCR) of mRNA were measured by using an MTT-base assay, caspase 3/7 Glo LUC reagent (Promega, Madison, WI, USA), soft agar colony formation assay, and SYBR premix Ex Taq (Takara, Dalian, China), respectively. All the methods involved are conventional ones, which are available elsewhere.

### Mass Spectrometry

To identify possible proteins interacting with LXN, FAU, RPL29, AHSG, ALB, and TF, respectively, the protein bands in the Coomassie Brilliant blue gel were excised and in-gel digested with trypsin. Then, the digests of the proteins were analyzed using a capillary electrophoresis/nano-liquid chromatography (Nano-LC) systems coupled with an electrospray ionization and time-of-flight mass spectrometer (ESI-QTOF-MS, Bruker Daltonics, Leipzig, Germany). An internal MASCOT 2.4.1 server (Matrix Science, Boston, MA, USA; http://www.matrixscience.com/) using the Swiss-Prot database was used to identify peptides.

### Protein Ligation Assay (PLA)

PLA was performed to identify the direct interaction between exogenous NMT1 and NUP/NDP using the Duolink In Situ Red Starter Kit (mouse/rabbit) (Sigma-Aldrich, Uppsala, Sweden). Briefly, cells were seeded on glass cover slips in 24-well plates and fixed with 4% paraformaldehyde. After blocking with the blocking buffer supplied by the manufacturer, cells were incubated overnight at 4°C in suitable primary antibodies (anti-FLAG, CST, #8146, and anti-HA, CST, #3724). Then, the PLA probe solution was added to cells followed by adding the ligase-ligase solution. After ligation, the amplification-polymerase solution was added to the cells for 100 min at 37°C and the cells were finally subjected to microscopic analysis. The bright fluorescent emissions can be detected if the proximity between the two PLA probes is < 40 nm.

### YnMyr Labeling and Copper-Catalyzed Azide-Alkyne Cycloaddition (CuAAC)

Cells were incubated with culture media containing the YnMyr (20 µM, WuXi AppTec, Shanghai, China) for 24 h before harvest. Then, a click mixture [mixed with the capture reagent AzTB (0.1 mM, WuXi AppTec), CuSO_4_ (1 mM), tris(2-carboxyethyl)phosphine (TCEP) (1 mM), and tris(benzyltriazolylmethyl)amine (TBTA) (0.1 mM) in DMSO] was added to each sample before vortexing at RT for 1 h. After adding 1 ml ice-cold MeOH containing EDTA (10 mM), the samples were quickly vortexed and kept at −80°C overnight. Next day, the samples were centrifuged to pellet precipitated proteins, and the pellets were dissolved by 75 µl 2% SDS in PBS and 25 µl 4 × SLB [sample-loading buffer prepared by mixing 4 × NuPAGE™ LDS sample buffer (Thermo Fisher Scientific, Inc. Wal-tham, MA, USA) and ß-mercaptoethanol of a ratio of 5:1]. Finally, the samples were subjected to WB using primary antibody against TAMRA (Abcam, #ab171120 or Invitrogen, #A6397).

### Motif Discovery

In order to reveal the NMT1-associated motif within NUP/NDP and the HIST1H4H-interacted motif within HBB and RPL7A, a protein sequences and homology analysis was conducted using conserved domains database (CDD, version 3.16), and Blastp. In order to find distinct related pattern, an e-value cutoff was set to 10 as default setting for short peptides (more than seven amino acids) mapping and the similarity cutoff was set to 30%. Linked Sequence alignment was performed with MUSCLE (version 3.8.31).

### Analysis of Ubiquitinase-Related Domain

Firstly, the protein sequences of the ubiquitin-related enzymes (totally 408 proteins were found in the Uniprot database) were extracted and downloaded from the Uniprot database. Then, the ubiquitinase domain sequence database was created by obtaining corresponding sequences from these 408 proteins. After that, the ubiquitinase-related domain database was aligned with the domain sequence library generated from the six candidate genes (data from **Figure 6A**) using the Blastp. Consequently, the blast results were screened using the evalue parameter cutoff of 0.5. The results predicted that the pfam02291 domain (a.a., 66–103) within the protein HIST1H4H (NP_003534) resembles the domain pfam13540 (a.a., 4293–4322) of the Ubiquitin E3 ligase HERC1 (NP_003913).

### *In Vitro* Ubiquitination Assays

Purified E1, E2 (UBE2S), and Ub were purchased from Boston Biochemistry (Cambridge, MA, USA). ATP was purchased from Sigma (St. Louis, MO, USA). Purified HIST1H4H, LXN, RPL29, FAU, and AHSG were purchased from Abnova (Taiwan, China). TF and ALB were purchased from USBiological (Swampscott, MA, USA). Briefly, E1 (50 ng), E2 (200 ng), and Ub (5 µg), NDP/NUP (500 ng) were incubated with or without HIST1H4H (300 ng) in 50 µl reaction buffer (0.05 M Tris-HCl (pH 7.5), 2 mM ATP, 5 mM MgCl_2_, and 2 mM DTT) at 30°C for 30 min, and the assay was terminated with protein loading buffer (Beyotime) and boiled at 100°C for 10 min before subjecting to WB using the corresponding antibodies [anti-ALB (Arigo, #ARG54036), anti-TF (Abcam, #ab82411), anti-AHSG (Arigo, #ARG55425), anti-LXN (Abcam, #ab154744), anti-RPL29 (Abcam, #ab67196), and anti-FAU (Abcam, #ab63065), anti-Ub (CST, #3936 or #3933)].

### Parallel Reaction Monitoring (PRM) and Isobaric Tags for Relative and Absolute Quantification (iTraq)

The PRM was performed by PTM BIO (Hangzhou, China). Briefly, the differential expression of selected proteins was detected using a Q-exactive mass spectrometer (Thermo Fisher Scientific, Bremen, Germany). After protein extraction and trypsin digestion, the tryptic peptides were prepared using an EASY-nLC 1000 UPLC system. And the resulting MS data were processed using Skyline software (v.3.6). The iTraq was performed by Luming Biotechnology (Shanghai, China). Briefly, peptides were labeled using an iTraq 8-plex kit (AB SCIEX, Washington, DC) in two technical replicates as per manufacturer’s instructions. The iTraq-based LC-MS/MS analysis was carried out using a Triple TOF 5600 System (AB SCIEX, USA) fitted with a Nanospray III source (AB SCIEX, USA) and a pulled quartz tip as the emitter (New Objectives, USA). Data were processed by the protein pilot software (v. 5.0, AB SCIEX, USA) against Homo Sapiens database using the Paragon algorithm.

### Statistical Analysis

The survival analysis was performed using the Kaplan-Meier curves and the log-rank test. χ^2^ test and one-way ANOVA were used to examine the differences between groups. A *p* < 0.05 was considered statistically significant.

### Data Availability

PRM, MS, and iTraq data have been deposited in the ProteomeXchange Consortium *via* the PRIDE partner repository under accession code PXD011764 (PRM), PXD011906 (MS), and PXD011812 (iTraq). The authors declare that all the data supporting the findings of this study are available within the article and its **Supplementary Information Files**, or available from the authors on request.

## Results

### NMT1 and N-myristoylation Boost Transformative Phenotypes

**Figure 1:**
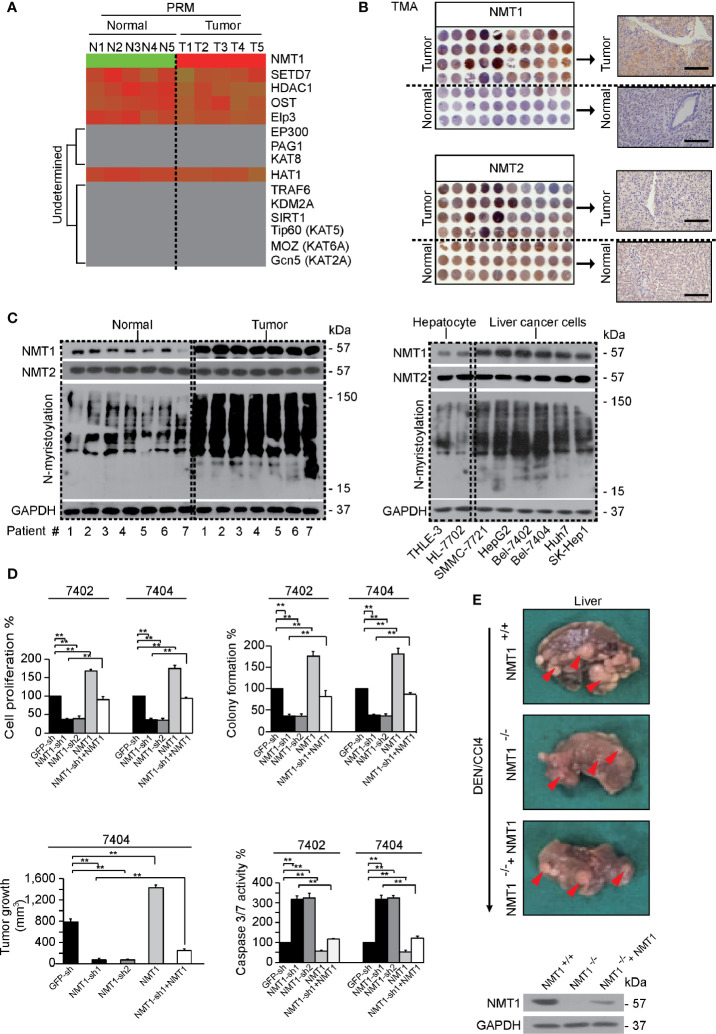
NMT1 and N-myristoylation were critical for liver cancer **(A)** NMT1 was identified highly elevated in liver cancer. Five pairs tumor-normal liver specimen were subjected into the PRM experiment, and 15 protein modification enzymes including NMT1 were evaluated. **(B)** The expression of NMT1 and NMT2 was analyzed by TMA using IHC. The representative IHC images of tumor and normal liver tissues are shown on the right. Scale bar, 200 μm. **(C)** Representative WB images of NMT1, NMT2 and global N-myristoylation in tumor and normal liver tissues from 7 patients (left). The expression pattern of the same proteins is also shown in established hepatocyte and liver cancer cell lines (right). **(D)** NMT1 was critical for maintaining transformative phenotypes in liver cancer cell lines, Bel-7402 and Bel-7404. Capacities of cell proliferation, colony formation, and *in vivo* tumor growth as well as Caspase 3/7 activity were measured by an MTT-based proliferation assay, soft agar colony formation assay, xenograft assay and Caspase 3/7 Glo reagent, respectively, in control cells, Bel-7402 or Bel-7404 cells with NMT1 knocked down or overexpressed. **(E)** NMT1 was essential for DEN/CCl4-induced liver tumorigenesis. The liver from control mice (NMT1^+/+^), NMT1 liver conditional knock-out mice (NMT1^−/−^) and NMT1 liver conditional knock-out mice injected with plasmids expressing exogenous NMT1 (NMT1^−/−^+NMT1) are shown after DEN/CCl4 induction (upper). The representative tumor foci are indicated by red arrows. The protein levels of NMT1 were also measured by WB (lower), n=5/group. The data are shown as the means + SD from 3 independent experiments (**Figure 1D** except xenograft experiments). Images of WB are representative ones of 3 independent experiments. **p < 0.01 indicates statistical significance. The data from **Figure 1D** were analyzed by a one-way ANOVA test.

We performed Parallel reaction monitoring (PRM) to evaluate the expression of 15 well-acknowledged protein modification enzymes in five pairs of liver cancer and normal liver tissues. Only NMT1 was identified highly up-regulated in liver cancer compared to normal liver (**Figure 1A**). Unfortunately, several enzymes could not be determined (**Figure 1A**), which might be due to the low abundance of these enzymes. *Via* tissue microarray assay (TMA), we further confirmed that NMT1 but not NMT2, another NMT member, was elevated in liver cancer (**Figure 1B**). Moreover, the levels of global N-myristoylation and NMT1 but not NMT2 were parallel elevated not only in clinical liver cancer specimen but also in established liver cancer cell lines (SMMC-7721, HepG2, Bel-7402, Bel-7404, Huh7, and SK-Hep1) compared to normal liver tissues and established hepatocyte lines (THLE-3 and HL-7702), respectively (**Figure 1C**). As Bel-7402 and Bel-7404 were two cell lines with the highest levels of NMT1 and N-myristoylation (**Figure 1C**), we chose these two as the main materials.

Next, we investigated whether NMT1 and N-myristoylation are critical for liver tumorigenesis. We found that both the levels of NMT1 and global N-myristoylation were successfully suppressed or stimulated by knocking or overexpressing NMT1 (**Supplementary Figure S1A**). Further, knocking NMT1 down significantly impaired transformative phenotypes including reduced cell proliferation, colony formation, and *in vivo* tumor growth capacities whereas induced caspase 3/7 activity. By contrast, overexpressing NMT1 led to the opposite effects. All the effects resulted from knocking NMT1 down could be reversed by simultaneously overexpressing NMT1 (**Figure 1D** and **Supplementary Figures S1B, C**). Also, by liver conditionally knocking NMT1 out in mice (NMT1^−/−^, NMT1^flox/flox^: Alb-*Cre*), we observed that treating DEN/CCl4 was unable to cause obvious formation of tumor loci compared to the liver of control mice (NMT1^+/+^, Alb-*Cre*). Via tail injecting of liver specific NMT1 expressing plasmids in NMT1 knock-out mice, we generated the NMT1 rescue mice (NMT1^−/−^+NMT1, NMT1^flox/flox^: Alb-*Cre*+NMT1), and expectedly, larger and more tumor loci could be detected in the liver of this kind of mice compared to the NMT1 knock-out mice (**Figure 1E**), suggesting liver tumorigenesis is indispensible of NMT1.

We also excluded the possibility that the effect of NMT1 depends on NMT2, because simultaneously overexpressing NMT2 was unable to rescue down-regulation of NMT1 and impaired transformative phenotypes caused by knocking NMT1 down (**Supplementary Figures S1D, E**).

### Proteins With Two Different Motifs Can Be N-myristoylated

**Figure 2:**
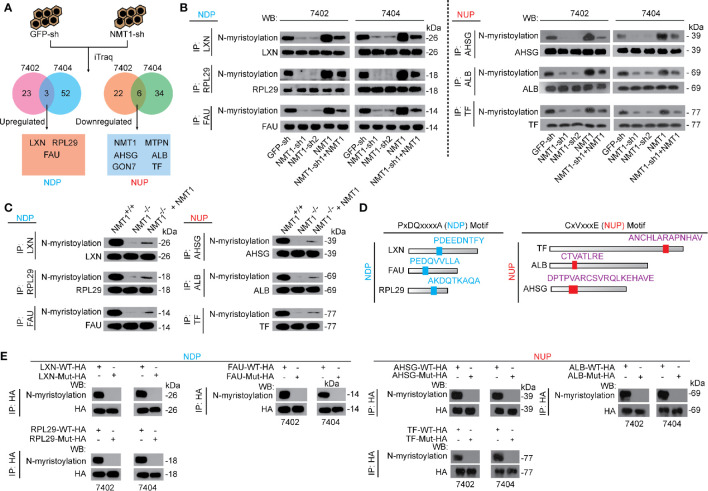
Two categories of NMT1 targets could all be N-myristoylated. **(A)** NDP and NUP were identified up-regulated or down-regulated by NMT1 knockdown in both Bel-7402 and Bel-7404 cells, as revealed by iTraq (two biological replicates). **(B)** N-myristoylation of both NDP and NUP could be positively regulated by NMT1. NDP and NUP were immuno-precipitated by corresponding antibodies and visualized by CuAAC and WB in both Bel-7402 and Bel-7404 cells. **(C)** N-myristoylation of NDP and NUP were positively controlled by NMT1 in mouse liver. NDP and NUP were immuno-precipitated from liver of mice, as indicated, and N-myristoylation of NDP and NUP were measured by CuAAC and WB. **(D)** Motif discovery of common motifs in NDP and NUP. **(E)** NDP and NUP motifs were critical for N-myristoylation of NDP and NUP. Exogenous NDP/NUP with (WT) or without functional NDP/NUP motifs (Mut) were expressed in Bel-7402 and Bel-7404 cells. N-myristoylation of exogenous NDP/NUP were immuno-precipitated by anti-HA antibodies and measured by CuAAC and WB. Images of WB are representative ones of 3 independent experiments.

We performed proteomics isobaric tags for relative and absolute quantification (iTraq) to identify proteins that changed before and after NMT1 was knocked down. Three proteins, namely Latexin (LXN), ribosomal protein L29 (RPL29), and ribosomal protein S30 (FAU), were identified up-regulated while six proteins, NMT1 per se, Myotrophin (MTPN), Alpha 2-HS glycoprotein (AHSG), Albumin (ALB), EKOPS complex subunit GON7 (GON7), and Serotransferrin (TF), were identified down-regulated after NMT1 was knocked down in both Bel-7402 and Bel-7404 cells (**Figure 2A**). We named these proteins as N-myristoylation down-regulated protein (NDP) and up-regulated protein (NUP), respectively.

Moreover, only protein but not mRNA levels of NDP and NUP can be altered by NMT1 (**Supplementary Figures S2A,B**). Also, we confirmed that NDP could be up-regulated whereas NUP could be down-regulated by knocking NMT1 down, and such effects could be reversed by simultaneously overexpressing NMT1 (**Supplementary Figure S2B**). Because the changes of GON7 and MTPN were not as significant as those of AHSG, ALB, and TF, we thereby only focused on AHSG, ALB, and TF in NUP (**Supplementary Figure S2B**). By contrast, overexpressing NMT1 indeed suppressed NDP while it stimulated NUP (**Supplementary Figure S2B**). The relationships among NDP, NUP, and NMT1 were also validated in mouse models (**Supplementary Figure S2C**).

Interestingly, knocking down NMT1 reduced whereas overexpressing NMT1 induced N-myristoylation of both NDP and NUP (**Figure 2B**). In mouse models, knocking NMT1 out caused de-N-myristoylation of both NDP and NUP, which could be partially reversed by expressing exogenous NMT1 in the liver (**Figure 2C**). These findings demonstrate that although NMT1 plays opposite roles on the expression of NDP and NUP, NMT1 only stimulates N-myristoylation of both NDP and NUP.

Then, we tried to reveal similarities among NDP and among NUP. A PxDQxxxxA (hereafter named as the NDP motif) within the LXN, FAU, and RPL29, and a CxVxxxE (hereafter named as NUP motif) within the TF, ALB, and AHSG were revealed (**Figure 2D**). To evaluate the function of these two motifs, we generated WT and mutant-NDP and -NUP expressing constructs, and found that without the functional NDP and NUP motifs, NDP and NUP could not be N-myristoylated (**Figure 2E**).

### NMT1 Regulates Protein Stability *via* the NDP and NUP Motifs

**Figure 3:**
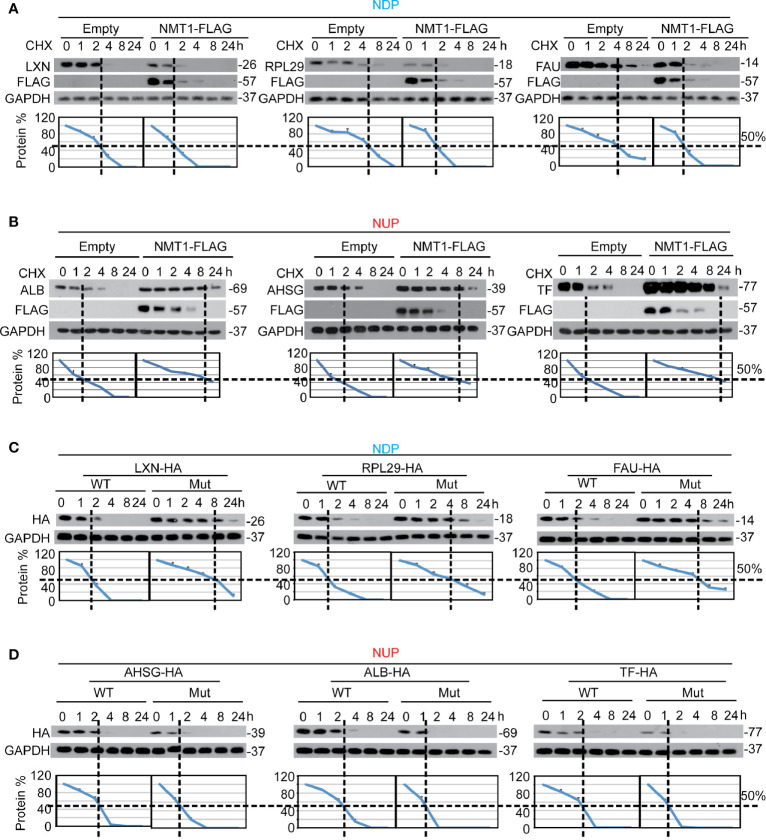
NMT1 oppositely regulated half-life of NDP/NUP. **(A, B)** NMT1 shortened half-life of NDP while prolonged half-life of NUP. Protein was harvested in Bel-7402 cells with or without NMT1 overexpression at indicated time after adding CHX (final concentration 0.1mg/ml). WB was then performed to evaluate half-life of NDP **(A)** and NUP **(B)**, respectively. **(C, D)** The NDP and NUP motifs were critical for the half-life of NDP and NUP. CHX chase experiments were performed in Bel-7402 cells expressing exogenous NDP/NUP with (WT) or without (Mut) functional NDP/NUP motifs. The relative protein levels were graphed as the levels of NDP/NUP to the levels of GAPDH from 3 independent experiments, and the relative levels of “0 h” were arbitrarily set to 100%. Images of WB are representative ones of 3 independent experiments.

Because protein modification, such as phosphorylation and O-GlcNAcylation, affect protein expression *via* influencing their stability, we postulated that N-myristoylation and NMT1 also affects protein stability. We found that overexpression of NMT1 led to a shortened half-life of NDP **(** **Figure 3A**), whereas a prolonged half-life of NUP (**Figure 3B**). However, NMT1 was unable to do so once the NDP and NUP motifs were mutated (**Supplementary Figures S3A, B**). Then, we compared the protein half-life between exogenous WT and mutant NDP and NUP. Expectedly, compared to the WT one, mutant NDP had a longer half-life **(** **Figure 3C**), whereas mutant NUP had a shorter one (**Figure 3D**). These data are indicative of the importance of NMT1 in the opposite regulation of protein stability possibly *via* the NDP and NUP motifs.

Further, we found that mutation of the NDP and NUP motifs abolished NMT1 interaction with NDP and NUP (**Supplementary Figures S3C, D**), suggesting the NDP and NUP motifs are also essential for the interaction between NMT1 and its target.

### POTEE Is Essential for NMT1-Mediated N-myristoylation

**Figure 4:**
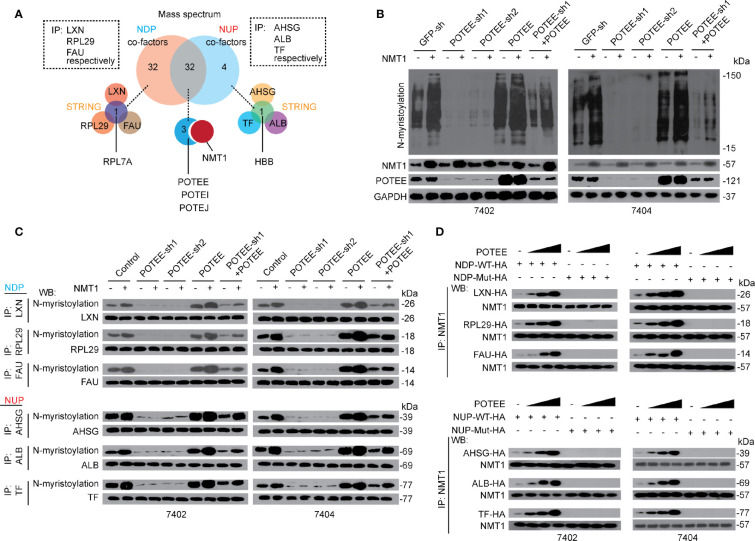
POTEE was essential for NMT1-mediated N-myristoylation. **(A)** RPL7A, HBB and POTE family were identified specifically and commonly interacted with NDP and NUP. Three NDP (including LXN, RPL29 and FAU) and three NUP (including AHSG, TF and ALB) were immuno-precipitated by their corresponding antibodies, respectively. The immuno-precipitates were then subjected into MS and the data (two biological replicates) were further evaluated by software STRING. **(B)** NMT1 stimulated global N-myristoylation depended on POTEE. Global N-myristoylation were measured by CuAAC in control cells, Bel-7402 and Bel-7404 cells with or without POTEE knocked down or overexpressed, as indicated. **(C)** POTEE was essential for N-myristoylation of both NDP and NUP. N-myristoylation of NDP and NUP was measured by CuAAC and WB in the immuno-precipitates that were immuno-precipitated by the corresponding antibodies, as indicated, in Bel-7402 and Bel-7404 cells. **(D)** POTEE was critical for the interaction between NMT1 and NDP/NUP. Endogenous NMT1 was immuno-precipitated by anti-NMT1 antibodies in Bel-7402 and Bel-7404 cells overexpressing exogenous WT (with functional NDP/NUP motif) or Mutant (without functional NDP/NUP motif) NDP/NUP-HA. The cells were also transfected with or without increasing concentration of POTEE expressing plasmids. Images of WB are representative ones of 3 independent experiments.

Next, we investigated the potential factors contributing to control of N-myristoylation and expression of NDP and NUP. We immuno-precipitated NDP including LXN, RPL29, and FAU, respectively, and NUP including AHSG, ALB, and TF, respectively, before setting the immuno-precipitates to the Mass spectrometry (MS). Thirty-two proteins were identified only co-interacted with the NDP, while four proteins were identified only co-interacted with the NUP. Moreover, 32 proteins were common ones co-interacted with both NDP and NUP (**Figure 4A**). To narrow potential candidates that co-interact with NDP and NUP, Search Tool for the Retrieval of Interacting Genes/proteins (STRING) was performed, and revealed that only RPL7A co-interacted with NDP, and only HBB co-interacted with NUP (**Figure 4A** and **Supplementary Figures S4A, B**). Interestingly, three POTE members, i.e. POTEE, POTEI and POTEJ, were also identified to co-interact with NMT1 (**Figure 4A** and **Supplementary Figure S4C**). *Via* public available data (BioProject: PRJEB4337), we noticed that only POTEE was obviously expressed in the liver (**Supplementary Figure S4C**); thereby we mainly focused on POTEE.

Then we investigated whether POTEE is essential for NMT1-mediated N-myristoylation. We found that global N-myristoylation was significantly reduced when POTEE was knocked down, while it was induced when POTEE was overexpressed. Moreover, compared to the control, the effects that NMT1-induced elevation of global N-myristoylation was almost blocked in cells with POTEE knocked down, which could be reversed by simultaneously overexpressing POTEE. By contrast, overexpressing POTEE enhanced the effects by NMT1 (**Figure 4B**). Besides global N-myristoylation, we also evaluated specific N-myristoylation of NDP and NUP, respectively. Like global N-myristoylation, NMT1-mediated N-myristoylation of both NDP and NUP was indispensible for POTEE (**Figure 4C**). These results demonstrate that POTEE boosts the function of NMT1 in stimulating N-myristoylation.

Further, POTEE was able to enhance the interaction between NMT1 and NDP or NUP does-dependently. However, overexpressing POTEE was still unable to recover the interaction between NMT1 and NDP or NUP when the critical NDP or NUP motifs were mutated (**Figure 4D**). These data indicate that POTEE might exert its positive role *via* reinforcing the interaction between NMT1 and its targets through the NDP and NUP motifs.

### RPL7A and HBB Regulates NDP/NUP Expression *via* Affecting Ubiquitination

**Figure 5:**
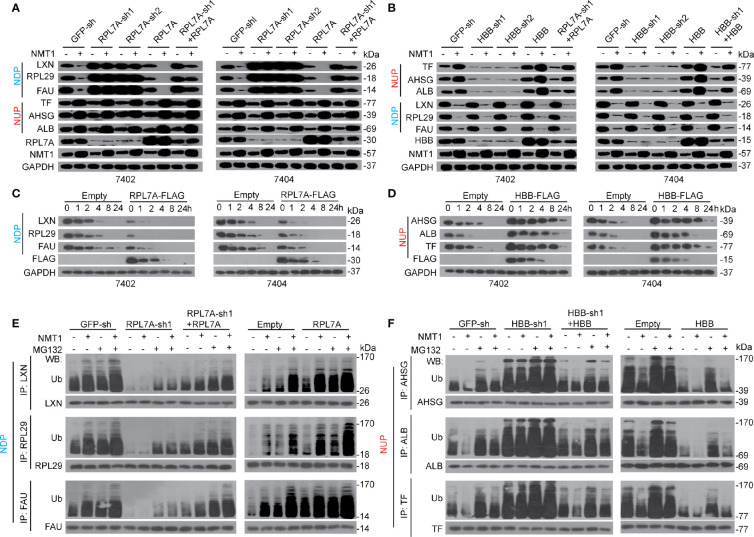
RPL7A/HBB controlled NDP/NUP expression *via* ubiquitination. **(A)** RPL7A was essential for NMT1-reduced NDP. The expression of NDP/NUP in control cells, Bel-7402 and Bel-7404 cells with RPL7A knocked down or overexpressed, in the absence or presence of NMT1 overexpression, as measured by WB. **(B)** HBB was essential for NMT1-indudced NUP. The expression of NUP/NDP in control cells, Bel-7402 and Bel-7404 cells with HBB knocked down or overexpressed, in the absence or presence of NMT1 overexpression, as measured by WB. **(C, D)** RPL7A and HBB oppositely regulated half-life of NDP and NUP. CHX-chase experiments were performed in Bel-7402 and Bel-7404 cells with or without overexpression of RPL7A **(C)** or HBB **(D)**. **(E, F)** RPL7A/HBB oppositely controlled ubiquitination of NDP/NUP. Ubiquitination of NDP **(E)** and NUP **(F)** in Bel-7402 cells was measured by firstly immuno-precipitation of NDP/NUP by corresponding antibodies, as indicated, followed by WB using anti-Ub antibodies. Cells with or without knocked down or overexpressed with RPL7A **(E)** or HBB **(F)** were simultaneously treated with or without overexpression of NMT1. MG132 was treated at a final concentration of 25µM. Images of WB are representative ones of 3 independent experiments.

Despite POTEE positively affecting N-myristoylation of both NDP and NUP, NMT1 and N-myristoylation cause opposite outcomes of NDP and NUP. Moreover, NDP and NUP specifically bind with RPL7A and HBB. Thereby, we hypothesized that NMT1 and N-myristoylation oppositely regulate expression of NDP and NUP might *via* RPL7A and HBB, respectively. We observed that the effects resulted from NMT1 overexpression, which caused down-regulation of NDP while up-regulation of NUP, were almost abolished when RPL7A or HBB was knocked down. Such effects could be reversed by simultaneously overexpressing RPL7A or HBB (**Figures 5A, B** **)**. By contrast, overexpressing RPL7A or HBB reinforced NMT1 to suppress or stimulate NDP or NUP (**Figures 5A, B** **)**. However, RPL7A specifically affected NDP but not NUP, and vice versa for HBB (**Figures 5A, B** **)**. Interestingly, neither RPL7A nor HBB influenced N-myristoylation (**Supplementary Figures S5A, B**). These data suggest that RPL7A and HBB might be merely essential for NMT1-mediated expression regulation of targets, but not necessary for NMT1-mediated N-myristoylation.

Because NMT1 affects protein stability of NDP and NUP, we postulated that RPL7A and HBB might be important for this process. Expectedly, overexpressing RPL7A resulted in a shortened half-life of NDP (**Figure 5C**). By contrast, overexpressing HBB led to a prolonged half-life of NUP (**Figure 5D**). Further, knocking down RPL7A and HBB led to the opposite outcome (**Supplementary Figures S5C, D**).

We also found that knocking RPL7A down not only reduced ubiquitination of NDP at basal level, but also blocked further ubiquitination of NDP by NMT1, and by contrast, overexpressing RPL7A led to the opposite outcome (**Figure 5E**). As for HBB, knocking down this protein induced ubiquitination of NUP at basal level, and blocked further suppression of ubiquitination by NMT1. By contrast, overexpressing HBB caused opposite effects (**Figure 5F**). Besides, ubiquitination of NDP and NUP could be reinforced by MG132, an inhibitor of proteasome; thereby, this further confirmed that NMT1 regulates expression of NDP and NUP *via* a ubiquitin-proteasome system. Collectively, the above results suggest that NMT1 regulates NDP and NUP expression possibly *via* affecting ubiquitination by RPL7A and HBB.

### RPL7A and HBB Regulates Ubiquitination *via* HIST1H4H

**Figure 6:**
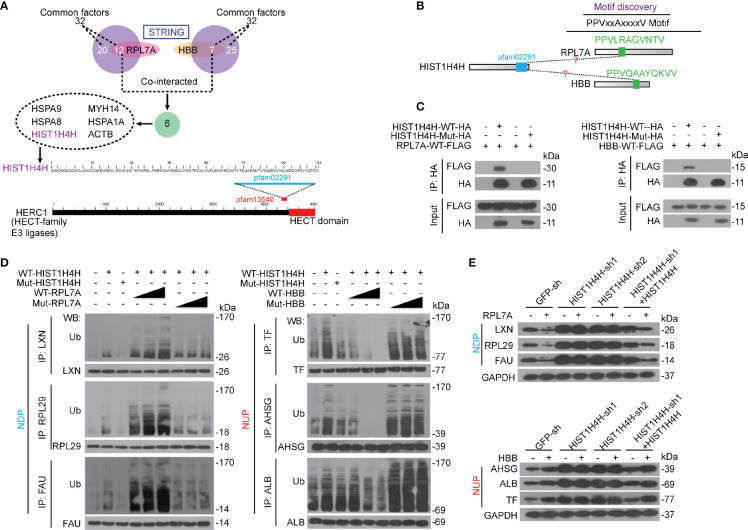
RPL7A and HBB regulated NDP and NUP *via* HIST1H4H. **(A)** HIST1H4H was predicted to co-interact with RPL7A and HBB. STRING was performed to reveal the interactions among HIST1H4H, RPL7A and HBB. The domain within HIST1H4H was aligned using Blastp, and the data were extracted from Uniprot. **(B)** The PPVxxAxxxxV motif was predicated to be similar between RPL7A and HBB, and the bioinformatics was conducted using the CDD database and Blastp. **(C)** RPL7A and HBB interacted with HIST1H4H. WT or mutant HIST1H4H (without functional pfam02291), as indicated, was co-expressed with WT RPL7A and HBB, respectively in Bel-7402 cells. The exogenous HIST1H4H-HA was immuno-precipitated by anti-HA antibodies, and co-immuno-precipitation of exogenous RPL7A-FLAG and HBB-FLAG was visualized by WB using anti-FLAG antibodies. **(D)** RPL7A and HBB oppositely controlled ubiquitination of NDP and NUP that mediated by HIST1H4H. Bel-7402 cells were co-overexpressed with WT or mutant HIST1H4H (without functional pfam02291) and WT or mutant RPL7A/HBB (without functional PPVxxAxxxxV motif), as indicated. Ubiquitination of NDP/NUP was firstly immuno-precipitation of NDP/NUP by the corresponding antibodies, and then visualized by anti-Ub antibodies using WB. **(E)** RPL7A and HBB regulate NDP and NUP expression *via* HIST1H4H. The protein expression of NDP and NUP were measured by WB in Bel-7402 cells with or without HIST1H4H knocked down or overexpressed, in the presence or absence of RPL7A (upper) or HBB (lower) overexpression. Images of WB are representative ones of 3 independent experiments.

Since both RPL7A and HBB have not been reported to be directly linked to ubiquitination, we then investigated potential factors contribute to the ubiquitination of NDP and NUP. STRING was performed to identify potential interactions, and six factors, namely heat shock protein family A member 9 (HSPA9), myosin heavy chain 14 (MYH14), heat shock protein family A member 8 (HSPA8), heat shock protein family A member 1A (HSPA1A), histone cluster 1 H4 family member H (HIST1H4H), and actin beta (ACTB), which all belong to the 32 common factors that have been identified to co-interact with both NDP and NUP (**Figure 4A**), were found to co-interact with both RPL7A and HBB (**Figure 6A**). Notably, only HIST1H4H was predicted to have a motif (pfam02291) that resembled the pfam13540 motif, the location of which is very close to the E3 ligase HECT domain of HECT and RLD domain containing E3 ubiquitin protein ligase family member 1 (HERC1). HERC1 is a well-established ubiquitin E3-ligase that belongs to the HECT family. Then, we investigated whether HIST1H4H acts as a genuine E3-ligase. *In vivo* cell-based ubiquitination experiments demonstrated that knocking HIST1H4H down led to de-ubiquitination of both NDP and NUP (**Supplementary Figure S6A**). *Via in vitro* ubiquitination experiments, we also found that both purified NDP and NUP could be ubiquitinated by HIST1H4H (**Supplementary Figure S6B**). These results suggested that HIST1H4H acts as a genuine E3 ligase to ubiquitinate NDP and NUP.

Via motif discovery, one PPVxxAxxxxV motif was identified in both RPL7A and HBB (**Figure 6B**). By co-IP experiments, the interaction between exogenous HIST1H4H and RPL7A was confirmed; however, when the pfam02291 motif of HIST1H4H was mutated, the interaction between HIST1H4H and RPL7A was blocked (**Figure 6C**), and vice versa for the PPVxxAxxxxV motif in RPL7A (**Supplementary Figure S6C**). Expectedly, the pfam02291 motif of HIST1H4H and the PPVxxAxxxxV motif of HBB are critical for the interaction between HIST1H4H and HBB (**Figure 6C** and **Supplementary Figure S6C**).

Next, we investigated the roles of HIST1H4H in the regulation of ubiquitination of NDP and NUP. We found that overexpressing HIST1H4H led to an elevated ubiquitination of both NDP and NUP; however, such effects were abolished when the pfam02291 motif was mutated (**Figure 6D**). Furthermore, overexpressing HIST1H4H-enhanced ubiquitination of NDP could be stimulated dose-dependently by RPL7A, yet mutation of the PPVxxAxxxxV motif in RPL7A had no such effects (**Figure 6D**). By contrast, HBB reduced ubiquitination of NUP by HIST1H4H dose-dependently; however, HBB without the PPVxxAxxxxV motif was unable to do so (**Figure 6D**). Also, we tested the influence of HIST1H4H, RPL7A, and HBB on the expression of NDP and NUP. We found that in control cells, overexpressing RPL7A suppressed expression of NDP, which could be almost abolished when HIST1H4H was knocked down, but was reversed when HIST1H4H was restored (**Figure 6E**). Similarly, knocking HIST1H4H down blocked HBB-induced elevation of NUP, which could also be restored by overexpressing HIST1H4H (**Figure 6E**). These findings suggest that RPL7A and HBB might exert their functions *via* affecting HIST1H4H-mediated ubiquitination of NDP and NUP.

### NMT1 Triggers RPL7A and HBB to Control HIST1H4H

**Figure 7:**
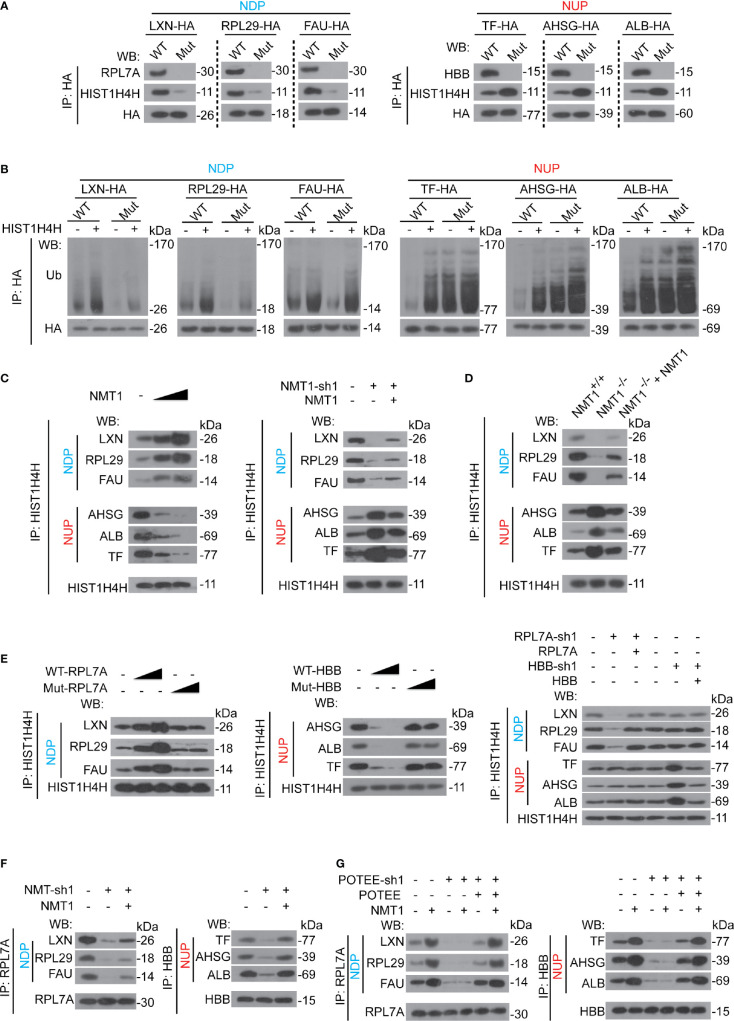
NMT1 regulated RPL7A and HBB to control HIST1H4H. **(A)** The NDP/NUP motifs were critical for RPL7A, HBB and HIST1H4H binding. NDP/NUP with (WT) or without (Mut) functional NDP/NUP motifs were immuno-precipitated by anti-HA antibodies in Bel-7402 cells, and co-immuno-precipitations of RPL7A, HBB and HIST1H4H were measured by indicated antibodies, as indicated. **(B)** Ubiquitination of NDP/NUP by HIST1H4H was NDP/NUP motif-dependent Ubiquitination of NDP/NUP was measured by firstly immuno-precipitation of NDP/NUP by anti-HA antibodies, followed by WB using anti-Ub antibodies in Bel-7402 cells expressing NDP/NUP with (WT) or without (Mut) NDP/NUP motifs in the absence or presence of HIST1H4H. **(C)** NMT1 oppositely controlled HIST1H4H binding to NDP/NUP. HIST1H4H was immuno-precipitated by anti-HIST1H4H antibodies in control cells, Bel-7402 cells expressing increasing concentration of NMT1 (left), and Bel-7402 cells with NMT1 knocked down with or without simultaneous overexpression of NMT1 (right). The co-immuno-precipitation of NDP and NUP was measured by WB using specific antibodies against NDP or NUP, as indicated. **(D)** The interaction between HIST1H4H and NDP/NUP was alerted when NMT1 was knocked out. The interaction between HIST1H4H and NDP/NUP in the liver of mice, as indicated, was measured using co-IP by firstly immuno-precipitation of HIST1H4H using anti-HIST1H4H antibodies followed by WB using anti-NDP/NUP antibodies, as indicated. **(E)** RPL7A and HBB are required for HIST1H4H binding to NDP/NUP. HIST1H4H was immuno-precipitated by anti-HIST1H4H antibodies in control cells, and Bel-7402 cells with RPL7A/HBB knocked down or ectopic expressed, as indicated. The co-immuno-precipitations of NDP and NUP were measured by antibodies, as indicated. **(F)** NMT1 is essential for RPL7A/HBB binding to NDP/NUP. RPL7A/HBB were immuno-precipitated in control cells, Bel-7402 cells with NMT1 knocked down or ectopic expressed, as indicated, and co-immuno-precipitations of NDP and NUP were measured by antibodies, as indicated. **(G)** POTEE was essential for NMT1-meidated RPL7A/HBB binding to NDP/NUP. RPL7A/HBB were immuno-precipitated by anti-RPL7A/HBB antibodies, and co-immuno-precipitation of NDP/NUP were measured by indicated antibodies in control cells, Bel-7402 cells with or without POTEE knocked down or ectopic expressed in the absence or presence of NMT1 overexpression. Images of WB are representative ones of 3 independent experiments.

We found that mutation of the NDP/NUP motifs in NDP/NUP blocked RPL7A/HBB binding to NDP/NUP (**Figure 7A**), suggesting besides being N-myristoylated, the NDP/NUP motifs are also important for RPL7A/HBB binding. Moreover, parallel to RPL7A, HIST1H4H dissociated away from NDP once the NDP motif was mutated. However, opposite to HBB, HIST1H4H recruited to NUP once the NUP motif was mutated (**Figure 7A**), indicating that the NDP motif is critical for recruiting HIST1H4H to NDP, while the NUP motif is essential for dissociating HIST1H4H from NUP.

Although HIST1H4H was still able to stimulate ubiquitination of NDP/NUP, regardless of the mutation of the NDP/NUP motifs or not, the overall ubiquitination levels of NDP/NUP were determined by the NDP/NUP motifs (**Figure 7B**), suggesting the NDP/NUP motifs might not be the recognition site of HIST1H4H, but instead to regulate basal HIST1H4H activity.

Because the NDP/NUP motifs can be N-myristoylated by NMT1, and are also essential to control HIST1H4H, we hypothesized that NMT1 might be the key to regulate HIST1H4H. We found that overexpressing NMT1 dose-dependently reinforced HIST1H4H binding to NDP, while suppressed HIST1H4H binding to NUP (**Figure 7C**). By contrast, knocking NMT1 down led to the opposite outcome (**Figure 7C**). Compared to the liver of WT mice, HIST1H4H was suppressed to bind with NDP, while was stimulated to bind to NUP in the mouse liver with NMT1 conditionally knocked out (**Figure 7D**). Expectedly, expressing exogenous NMT1 could reverse the effects by impaired expression of NMT1, either in human liver cancer cells or mouse liver (**Figures 7C, D** **)**.

Then, we investigated whether RPL7A and HBB are essential for HIST1H4H interacting with NDP/NUP. We found that RPL7A dose-dependently stimulated HIST1H4H binding to NDP, while HBB does-dependently suppressed HIST1H4H binding to NUP (**Figure 7E**). However, these effects disappeared when the PPVxxAxxxxV motif of RPL7A and HBB were mutated (**Figure 7E**). By contrast, knocking RPL7A down decreased HIST1H4H binding to NDP, whereas knocking HBB down increased HIST1H4H binding to NUP (**Figure 7E**). We also noticed that neither RPL7A nor HBB influenced HIST1H4H binding to NUP and NDP, respectively (**Figure 7E**), suggesting the effects of RPL7A and HBB on HIST1H4H might merely be NDP- and NUP-specific. Further, knocking NMT1 down directly inhibited RPL7A and HBB binding to NUP and NDP (**Figure 7F**), suggesting recruitment of RPL7A and HBB might also be controlled by NMT1.

Furthermore, overexpressing NMT1-caused recruitment of RPL7A to NDP, and HBB to NUP were abolished when POTEE was knocked down (**Figure 7G**). Expectedly, effects that resulted from knocking down POTEE could be reversed by ectopic expressing of POTEE (**Figure 7G**), suggesting the effects of NMT1 to regulate RPL7A and HBB interaction with NDP and NUP are indispensable to POTEE.

### Clinical Significance of NMT1, NDP, and NUP in Liver Cancer

Because NMT1 oppositely regulates the expression of NDP and NUP, and moreover, NMT1 has been proven to critically stimulate liver tumorigenesis, we hypothesized that NDP and NUP oppositely contribute to liver malignancy. Expectedly, knocking NDP down increased capacities of cell proliferation, colony formation, and *in vivo* xenograft growth, whereas it decreased Caspase 3/7 activity (**Supplementary Figures S7A–D**). By contrast, knocking NUP down resulted in the opposite outcome (**Supplementary Figures S7A–D**). These findings suggest that NDP and NUP might play opposite roles in maintaining transformative phenotypes.

**Figure 8:**
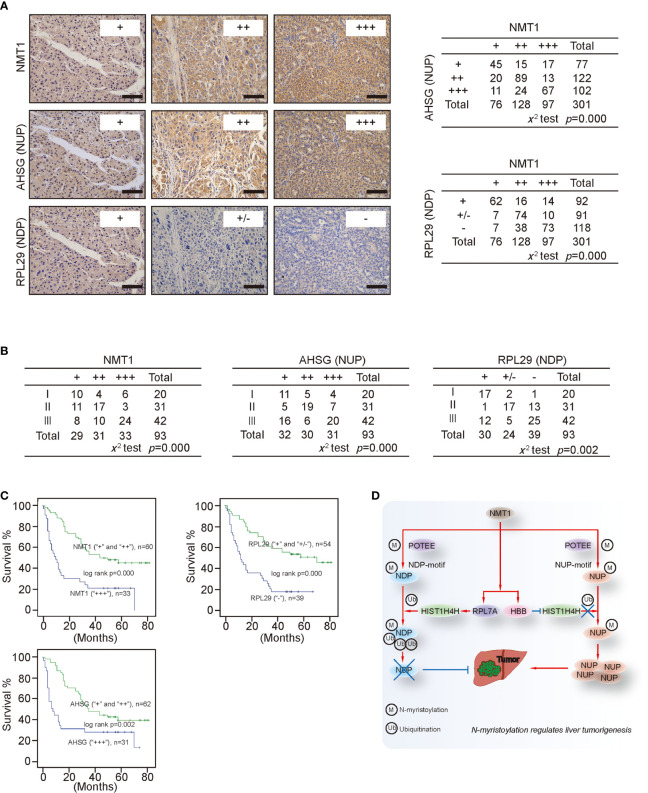
Clinical significance of NMT1, NDP and NUP **(A)** Representative IHC images of NMT1, AHSG and RPL29 from liver cancer are shown. The relationship between NMT1 and AHSG, and between NMT1 and RPL29 were evaluated by X^2^ test. Scale bar, 150μm **(B)** The relationship between tumor stage and NMT1, AHSG or RPL29 was evaluated in liver cancer by X^2^ test. **(C)** NMT1, RPL29 and AHSG affected survival. Patients bearing liver cancer with different expression of NMT1 (“+” and “++” versus “+++”), RPL29 (“+” and “+/-” versus “-”) and AHSG (“+” and “++” versus “+++”), as indicated, had different outcome of survival, as analyzed by the Kaplan-Meier curves and the log-rank test. **(D)** Schematic presentation of the possible mechanism underlying how NMT1 and N-myristoylation stimulate tumorigenesis in liver cancer.

Via iTraq, AHSG, and RPL29 were identified as the most up-regulated and down-regulated ones of the NUP and NDP we tested (**Supplementary Figure S7E**). Trough statistical analysis in 301 liver cancer specimens showed a positive relationship between AHSG and NMT1, whereas a negative relationship between RPL29 and NMT1 were revealed (**Figure 8A** **)**, further demonstrating that NMT1 might oppositely regulate expression of NUP and NDP. Moreover, both NMT1 and AHSG were associated with the tumor stage, while RPL29 reversely correlated with the tumor stage in liver cancer (**Figure 8B**). Notably, patients bearing liver cancer with higher levels of NMT1 and AHSG had a worse survival rate than those with lower level ones (**Figure 8C**). However, higher levels of RPL29 were indicative of a better survival (**Figure 8C**). These data suggest that NMT1, NUP, and NDP are closely associated with the clinical outcome in liver cancer.

## Discussion

Hepatocellular carcinoma (HCC) accounts for 85% to 90% of primary liver cancers, which remains the second leading cause of cancer-related death (19). In addition to Hepatitis B, metabolic disorders such as diabetes have also been identified as major risk factors for HCC (20). To date, the underlying mechanism of HCC development still remains largely unknown (21, 22). Thus, it is urgent to explore the oncogenes and potential treatment strategy. Recently, the pivotal role of PTMs during the carcinogenesis attracted increasing attentions. Typically, as one of the PTMs, sumoylation of certain proteins participated in the magnificent phenotype of HCC (23, 24). For instance, sumoylation of HIF-1α mediates its transcriptional activity and promotes angiogenesis of HCC (25). Moreover, targeting sumoylation synergize with FXR agonists in combating liver fibrosis (26). Exploring the potential role of PTMs may provide new view for HCC treatment.

In the current study, we have uncovered that NMT1 and possibly downstream N-myristoylation might recruit RPL7A and HIST1H4H to stimulate ubiquitination of NDP, while recruiting HBB to prevent HIST1H4H from binding and subsequently suppress ubiquitination of NUP. Thereby we established a regulatory model underlying how N-myristolyation up-regulates NUP whereas down-regulates NDP in liver cancer cells. We previously uncovered that occurrence of O-GlcNAcylation suppresses phosphorylation of specific target protein during initiation and progression of liver cancer (18). Here, we uncovered another important way to affect one kind PTM by another one, i.e. N-myristolation influences fate of target protein *via* interfering ubiquitination (**Figure 8D**).

Like the function of O-linked N-acetylglucosamine transferase (OGT) to O-GlcNAcylate targets (27), NMT1 directly N-myristolyates proteins. Although NMT1 recognizes two differential motifs located within the NDP and NUP, respectively, NDP and NUP can all be N-myristolyated. Furthermore, the function of NMT1 is POTEE-dependent, and POTEE is more likely to act as a co-factor. POTEE belongs to the POTE gene family that is consisted by 13 highly homologous variants, which are similarly made up of three distinct domains including ankyrin and spectrin (14), suggesting that they can interact with other regulatory proteins. Among members, POTEE is a dominant subtype that is expressed in many types of cancers and established cancer cell lines (28). Prior studies have shown that POTEE plays pivotal roles in the tumorigenesis originated from a number of organs, at least known in testis, ovary and prostate (29). However, POTEE is rarely studied in liver cancer. During our work, POTEE might provide a platform for NMT1 to interact with N-myristolyated substrates *via* its anchor mediated ligation. The mechanism underlying how POTEE interacts with NMT1 still needs to be further studied in the future.

Although both NDP and NUP can be N-myristoylated, the expression regulation outcomes of these two kinds of proteins by NMT1 are totally different. Such effects might be due to the opposite function of RPL7A and HBB. Both RPL7A and HBB are recruited to NDP and NUP once NDP/NUP are N-myristolyated, however, RPL7A reinforces ubiquitination of NDP, while HBB suppresses ubiquitination of NUP. To the best of our knowledge, RPL7A is a component of the 60s ribosomal proteins, its function is to catalyze protein biosynthesis (30). However, HBB is a subunit of hemoglobin (31), and its functions are unfortunately still unknown. Due to the regulatory roles of RPL7A and HBB are HIST1H4H-dependent, and HIST1H4H has been identified as a novel ubiquitin E3 ligase in the present study, we postulate that RPL7A and HBB might act as ubiquitin adaptor proteins. Interestingly, Tribbles pseudokinase 2 (TRIB2) has been reported to exert its roles assemble ubiquitin adaptor protein (32). TRIB2 regulates function of ubiquitin E3 ligases at least including β-transducin repeat containing E3 ubiquitin protein ligase (βTrCP) (33), however, TRIB2 either acts as a stimulator or suppressor to βTrCP possibly due to βTrCP targets, for example, TRIB2 prevents βTrCP to ubiquitinate YAP (32) while stimulates βTrCP to ubiquitinate TCF4 and βCatenin (34). Dislike TRIB2 controls a serial of ubiquitin E3 ligases, RPL7A, and HBB co-regulate a single ubiquitin E3 ligase. The pfam02291 domain of HIST1H4H resembles the pfam13540 domain, which is very close to the functional E3 ligase HECT domain in HERC1, and moreover, both the interaction between RPL7A and HIST1H4H, and between HBB and HIST1H4H all rely on the pfam02291, hence we speculate that the pfam02291 might act as a regulatory domain, by which RPL7A and HBB oppositely regulate HIST1H4H. Shao, et al. (35) have reported that E3 adaptor protein NUMB regulates HECT E3 ligase NEDD4-1 possibly *via* the regulatory domain WW, which supports our hypothesis that RPL7A and HBB act as E3 adaptors to regulate HIST1H4H. However, the mechanism underlying how RPL7A and HBB precisely regulate HIST1H4H remains unknown, and should be investigated in the near future.

Also, we identified that NDP are capable of inhibiting while NUP are capable of stimulating transformative phenotypes of liver cancer cells. Similar to our findings, Thompson et al. described that in AHSG, one protein belongs to NUP, has a role to promote tumorigenesis of head and neck squamous (36). Moreover, Zhao, et al., uncovered that AHSG is up-regulated in esophageal squamous cell carcinoma, and might also act as a pro-tumorigenic protein (37). By contrast, lower expression of RPL29, the one belongs to the NDP, is predicted to have a poorer outcome of unresectable pancreatic cancer (38), providing further evidence that RPL29 might be anti-tumorigenic. In liver cancer cells, we have revealed that NUPs are more likely to be predominantly expressed than NDPs, because NUPs are less ubiquitinated than regulated by NMT1. By contrast, NDPs can be diminished much faster by being ubiquitinated; thereby we have also explained one potential mechanism underlying how N-myristolyation promotes liver tumorigenesis by maintaining NUP expression while accelerating NDP degradation.

Previous studies reported YAP as a crucial oncogene in HCC development (39–41). Moreover, Andrea Bisso et al., demonstrated that YAP mediated the cooperation between MYC and β-Catenin and facilitated liver tumorigenesis (42). Wenbo Ma et al., also found that histone methyltransferase G9a promoted cholangiocarcinogenesis *via* YAP (43). Importantly, our previous work suggested that the function of YAP largely relied on its Thr241 O-GlcNAcylation (18). YAP O-GlcNAcylation enhanced protein stability by repressing its ubiquitination. Similarly, in the present study, N-myristolyation maintained NUP protein expression by repressing its ubiquitination and promoted HCC development, which further explain the underlying mechanism of liver tumorigenesis.

## Conclusion

A tremendous amount of studies have revealed that the PTMs of proteins are essential for tumorigenesis of liver cancer (44). Here, we have provided further evidence that N-myristolyation which is mediated by NMT1 plays critical roles in boosting liver tumorigenesis possibly *via* interfering the balance between anti-tumorigenic NDP and pro-tumorigenic NUP through controlling activity of ubiquitin E3 ligase HIST1H4H. Break N-myristolyation might be helpful to treat liver cancer.

## Data Availability Statement

The data sets presented in this study can be found in online repositories. The names of the repository/repositories and accession number(s) can be found in the article/**Supplementary Material**.

## Ethics Statement

The studies involving human participants were reviewed and approved by Shanghai Tenth People’s Hospital. The patients/participants provided their written informed consent to participate in this study. The animal study was reviewed and approved by Shanghai Tenth People’s Hospital.

## Author Contributions

GZ researched and analyzed data. HL, FW, XZ, SG, YY, XX, QW, YL, and FS researched the data. MQ collected and analyzed clinical samples. GZ and HL contributed to discussion. YQ and QP designed the study. YQ, HL, and GZ wrote the manuscript. All authors contributed to the article and approved the submitted version.

## Funding

This work was supported by the National Natural Science Foundation of China (grants 81872288, 81822029, 81871907 and 81672332), the Shanghai Rising-Star program (18QA1403400), and Shanghai Municipal commission of Health and Family Planning (key developing disciplines and outstanding youth training plan no. 2017YQ024).

## Conflict of Interest

The authors declare that the research was conducted in the absence of any commercial or financial relationships that could be construed as a potential conflict of interest.

## Abbreviations

PTMs, post-translational modifications; NMT, N-myristoyltransferase; CHX, Cycloheximide; qPCR, quantitative RT-PCR; PLA, protein ligation assay; CuAAC, Copper-catalyzed Azide-alkyne Cycloaddition; CDD, conserved domains database; PRM, parallel reaction monitoring; iTraq, isobaric tags for relative and absolute quantification; TMA, tissue microarray assay; NDP, N-myristoylation down-regulated protein; NUP, N-myristoylation up-regulated protein; MS, mass spectrometry; STRING, Search Tool for the Retrieval of Interacting Genes/proteins.

## References

[1] Xie Y Kang R Sun X Zhong M Huang J Klionsky DJ. Posttranslational Modification of Autophagy-Related Proteins in Macroautophagy. Autophagy (2015) 11(1):28–45. 10.4161/15548627.2014.984267 25484070 PMC4502723

[2] Wloga D Joachimiak E Louka P Gaertig J. Posttranslational Modifications of Tubulin and Cilia. Cold Spring Harb Perspect Biol (2017) 9(6).a028159 10.1101/cshperspect.a028159 28003186 PMC5453388

[3] Wang YQ Wang HL Xu J Tan J Fu LN Wang JL. Sirtuin5 Contributes to Colorectal Carcinogenesis by Enhancing Glutaminolysis in a Deglutarylation-Dependent Manner. Nat Commun (2018) 9(1):545. 10.1038/s41467-018-02951-4 29416026 PMC5803207

[4] Herhaus L Dikic I. Expanding the Ubiquitin Code Through Post-Translational Modification. EMBO Rep (2015) 16(9):1071–83. 10.15252/embr.201540891 PMC4576978 26268526

[5] Thinon E Morales-Sanfrutos J Mann DJ Tate EW. N-Myristoyltransferase Inhibition Induces ER-Stress, Cell Cycle Arrest, and Apoptosis in Cancer Cells. ACS Chem Biol (2016) 11(8):2165–76. 10.1021/acschembio.6b00371 PMC5077176 27267252

[6] Kim S Alsaidan OA Goodwin O Li Q Sulejmani E Han Z. Blocking Myristoylation of Src Inhibits Its Kinase Activity and Suppresses Prostate Cancer Progression. Cancer Res (2017) 77(24):6950–62. 10.1158/0008-5472.CAN-17-0981 PMC5732839 29038344

[7] Kosciuk T Price IR Zhang X Zhu C Johnson KN Zhang S. NMT1 and NMT2 are Lysine Myristoyltransferases Regulating the ARF6 Gtpase Cycle. Nat Commun (2020) 11(1):1067. 10.1038/s41467-020-14893-x 32103017 PMC7044312

[8] Wen Z Jin K Shen Y Yang Z Li Y Wu B. N-Myristoyltransferase Deficiency Impairs Activation of Kinase AMPK and Promotes Synovial Tissue Inflammation. Nat Immunol (2019) 20(3):313–25. 10.1038/s41590-018-0296-7 PMC6396296 30718913

[9] Thinon E Serwa RA Broncel M Brannigan JA Brassat U Wright MH. Global Profiling of Co- and Post-Translationally N-myristoylated Proteomes in Human Cells. Nat Commun (2014) 5:4919. 10.1038/ncomms5919 25255805 PMC4200515

[10] Dian C Perez-Dorado I Riviere F Asensio T Legrand P Ritzefeld M. High-Resolution Snapshots of Human N-myristoyltransferase in Action Illuminate a Mechanism Promoting N-terminal Lys and Gly Myristoylation. Nat Commun (2020) 11(1):1132. 10.1038/s41467-020-14847-3 32111831 PMC7048800

[11] Selvakumar P Lakshmikuttyamma A Shrivastav A Das SB Dimmock JR Sharma RK. Potential Role of N-myristoyltransferase in Cancer. Prog Lipid Res (2007) 46(1):1–36. 10.1016/j.plipres.2006.05.002 16846646

[12] Shrivastav A Varma S Senger A Khandelwal RL Carlsen S Sharma RK. Overexpression of Akt/PKB Modulates N-myristoyltransferase Activity in Cancer Cells. J Pathol (2009) 218(3):391–8. 10.1002/path.2550 19360752

[13] Shen Z Feng X Fang Y Li Y Li Z Zhan Y. POTEE Drives Colorectal Cancer Development *Via* Regulating SPHK1/p65 Signaling. Cell Death Dis (2019) 10(11):863. 10.1038/s41419-019-2046-7 31723122 PMC6853991

[14] Wang Q Li X Ren S Cheng N Zhao M Zhang Y. Serum Levels of the Cancer-Testis Antigen POTEE and Its Clinical Significance in Non-Small-Cell Lung Cancer. PloS One (2015) 10(4):e0122792. 10.1371/journal.pone.0122792 25860145 PMC4393100

[15] Bera TK Saint Fleur A Lee Y Kydd A Hahn Y Popescu NC. POTE Paralogs are Induced and Differentially Expressed in Many Cancers. Cancer Res (2006) 66(1):52–6. 10.1158/0008-5472.CAN-05-3014 16397215

[16] Tissir F Bar I Goffinet AM Lambert De Rouvroit C. Expression of the Ankyrin Repeat Domain 6 Gene (Ankrd6) During Mouse Brain Development. Dev Dyn (2002) 224(4):465–9. 10.1002/dvdy.10126 12203740

[17] Vekariya U Rawat K Saxena R Tripathi RK. Identification of MPhi Specific POTEE Expression: Its Role in mTORC2 Activation *Via* Protein-Protein Interaction in Tams. Cell Immunol (2019) 335:30–40. 10.1016/j.cellimm.2018.10.010 30420269

[18] Zhang X Qiao Y Wu Q Chen Y Zou S Liu X. The Essential Role of YAP O-GlcNAcylation in High-Glucose-Stimulated Liver Tumorigenesis. Nat Commun (2017) 8:15280. 10.1038/ncomms15280 28474680 PMC5424161

[19] Bian XL Chen HZ Yang PB Li YP Zhang FN Zhang JY. Nur77 Suppresses Hepatocellular Carcinoma *Via* Switching Glucose Metabolism Toward Gluconeogenesis Through Attenuating Phosphoenolpyruvate Carboxykinase Sumoylation. Nat Commun (2017) 8:14420. 10.1038/ncomms14420 28240261 PMC5333363

[20] Ren L Zeng M Tang Z Li M Wang X Xu Y. The Antiresection Activity of the X Protein Encoded by Hepatitis Virus B. Hepatology (2019) 69(6):2546–61. 10.1002/hep.30571 PMC6618260 30791110

[21] Ge C Vilfranc CL Che L Pandita RK Hambarde S Andreassen PR. The BRUCE-ATR Signaling Axis is Required for Accurate DNA Replication and Suppression of Liver Cancer Development. Hepatology (2019) 69(6):2608–22. 10.1002/hep.30529 PMC6541504 30693543

[22] Schneider AT Gautheron J Feoktistova M Roderburg C Loosen SH Roy S. Ripk1 Suppresses a TRAF2-Dependent Pathway to Liver Cancer. Cancer Cell (2017) 31(1):94–109. 10.1016/j.ccell.2016.11.009 28017612

[23] Qin G Tu X Li H Cao P Chen X Song J. Long Noncoding RNA P53-Stabilizing and Activating Rna Promotes P53 Signaling by Inhibiting Heterogeneous Nuclear Ribonucleoprotein K deSUMOylation and Suppresses Hepatocellular Carcinoma. Hepatology (2020) 71(1):112–29. 10.1002/hep.30793 31148184

[24] Liu J Wu Z Han D Wei C Liang Y Jiang T. Mesencephalic Astrocyte-Derived Neurotrophic Factor Inhibits Liver Cancer Through Small Ubiquitin-Related Modifier (SUMO)Ylation-Related Suppression of NF-kappaB/Snail Signaling Pathway and Epithelial-Mesenchymal Transition. Hepatology (2020) 71(4):1262–78. 10.1002/hep.30917 PMC7187412 31469428

[25] Li J Xu Y Long XD Wang W Jiao HK Mei Z. Cbx4 Governs HIF-1alpha to Potentiate Angiogenesis of Hepatocellular Carcinoma by Its SUMO E3 Ligase Activity. Cancer Cell (2014) 25(1):118–31. 10.1016/j.ccr.2013.12.008 24434214

[26] Zhou J Cui S He Q Guo Y Pan X Zhang P. Sumoylation Inhibitors Synergize With FXR Agonists in Combating Liver Fibrosis. Nat Commun (2020) 11(1):240. 10.1038/s41467-019-14138-6 31932588 PMC6957516

[27] Peng C Zhu Y Zhang W Liao Q Chen Y Zhao X. Regulation of the Hippo-YAP Pathway by Glucose Sensor O-Glcnacylation. Mol Cell (2017) 68(3):591–604.e5. 10.1016/j.molcel.2017.10.010 29100056

[28] Cine N Baykal AT Sunnetci D Canturk Z Serhatli M Savli H. Identification of ApoA1, HPX and POTEE Genes by Omic Analysis in Breast Cancer. Oncol Rep (2014) 32(3):1078–86. 10.3892/or.2014.3277 24969553

[29] Bera TK Walker DA Sherins RJ Pastan I. POTE Protein, a Cancer-Testis Antigen, is Highly Expressed in Spermatids in Human Testis and is Associated With Apoptotic Cells. Biochem Biophys Res Commun (2012) 417(4):1271–4. 10.1016/j.bbrc.2011.12.125 PMC3278037 22234308

[30] Zheng SE Yao Y Dong Y Lin F Zhao H Shen Z. Down-Regulation of Ribosomal Protein L7A in Human Osteosarcoma. J Cancer Res Clin Oncol (2009) 135(8):1025–31. 10.1007/s00432-008-0538-4 PMC12160200 19125294

[31] Maman S Sagi-Assif O Yuan W Ginat R Meshel T Zubrilov I. The Beta Subunit of Hemoglobin (Hbb2/Hbb) Suppresses Neuroblastoma Growth and Metastasis. Cancer Res (2017) 77(1):14–26. 10.1158/0008-5472.CAN-15-2929 27793844

[32] Dedhia PH Keeshan K Uljon S Xu L Vega ME Shestova O. Differential Ability of Tribbles Family Members to Promote Degradation of C/EBPalpha and Induce Acute Myelogenous Leukemia. Blood (2010) 116(8):1321–8. 10.1182/blood-2009-07-229450 PMC2938240 20410507

[33] Xu S Tong M Huang J Zhang Y Qiao Y Weng W. TRIB2 Inhibits Wnt/beta-Catenin/TCF4 Signaling Through Its Associated Ubiquitin E3 Ligases, Beta-Trcp, COP1 and Smurf1, in Liver Cancer Cells. FEBS Lett (2014) 588(23):4334–41. 10.1016/j.febslet.2014.09.042 25311538

[34] Wang J Park JS Wei Y Rajurkar M Cotton JL Fan Q. TRIB2 Acts Downstream of Wnt/TCF in Liver Cancer Cells to Regulate YAP and C/EBPalpha Function. Mol Cell (2013) 51(2):211–25. 10.1016/j.molcel.2013.05.013 PMC4007693 23769673

[35] Shao C Li Z Ahmad N Liu X. Regulation of PTEN Degradation and NEDD4-1 E3 Ligase Activity by Numb. Cell Cycle (2017) 16(10):957–67. 10.1080/15384101.2017.1310351 PMC5462079 28437168

[36] Thompson PD Sakwe A Koumangoye R Yarbrough WG Ochieng J Marshall DR. Alpha-2 Heremans Schmid Glycoprotein (AHSG) Modulates Signaling Pathways in Head and Neck Squamous Cell Carcinoma Cell Line SQ20B. Exp Cell Res (2014) 321(2):123–32. 10.1016/j.yexcr.2013.12.003 PMC4096485 24332981

[37] Zhao Y Wei L Shao M Huang X Chang J Zheng J. Brca1-Associated Protein Increases Invasiveness of Esophageal Squamous Cell Carcinoma. Gastroenterology (2017) 153(5):1304–19.e5. 10.1053/j.gastro.2017.07.042 28780075

[38] Muro S Miyake Y Kato H Tsutsumi K Yamamoto K. Serum anti-60S Ribosomal Protein L29 Antibody as a Novel Prognostic Marker for Unresectable Pancreatic Cancer. Digestion (2015) 91(2):164–73. 10.1159/000371545 25765324

[39] Weiler SME Pinna F Wolf T Lutz T Geldiyev A Sticht C. Induction of Chromosome Instability by Activation of Yes-Associated Protein and Forkhead Box M1 in Liver Cancer. Gastroenterology (2017) 152(8):2037–51.e22. 10.1053/j.gastro.2017.02.018 28249813

[40] Kim W Khan SK Gvozdenovic-Jeremic J Kim Y Dahlman J Kim H. Hippo Signaling Interactions With Wnt/beta-catenin and Notch Signaling Repress Liver Tumorigenesis. J Clin Invest (2017) 127(1):137–52. 10.1172/JCI88486 PMC5199712 27869648

[41] Cai WY Lin LY Hao H Zhang SM Ma F Hong XX. Yes-Associated Protein/TEA Domain Family Member and Hepatocyte Nuclear Factor 4-Alpha (HNF4alpha) Repress Reciprocally to Regulate Hepatocarcinogenesis in Rats and Mice. Hepatology (2017) 65(4):1206–21. 10.1002/hep.28911 27809333

[42] Bisso A Filipuzzi M Gamarra Figueroa GP Brumana G Biagioni F Doni M. Cooperation Between MYC and beta-Catenin in Liver Tumorigenesis Requires Yap/Taz. Hepatology (2020) 72(4):1430–43. 10.1002/hep.31120 31965581

[43] Ma W Han C Zhang J Song K Chen W Kwon H. The Histone Methyltransferase G9a Promotes Cholangiocarcinogenesis Through Regulation of the Hippo Pathway Kinase LATS2 and YAP Signaling Pathway. Hepatology (2020) 72(4):1283–97. 10.1002/hep.31141 PMC7384937 31990985

[44] Siddique HR Feldman DE Chen CL Punj V Tokumitsu H Machida K. NUMB Phosphorylation Destabilizes p53 and Promotes Self-Renewal of Tumor-Initiating Cells by a NANOG-dependent Mechanism in Liver Cancer. Hepatology (2015) 62(5):1466–79. 10.1002/hep.27987 PMC4618247 26174965

