# Supplementary material for: N-Myristoylation by NMT1 Is POTEE-Dependent to Stimulate Liver Tumorigenesis via Differentially Regulating Ubiquitination of Targets
Source: Front Oncol. 2021 May 31;11:681366. doi: 10.3389/fonc.2021.681366 (PMC8201403; doi:10.3389/fonc.2021.681366)
Supplement: Supplementary file 1 [file DataSheet_1.doc]

**Supplementary Figures. S1-7**

**Supplementary Figure. S1.**


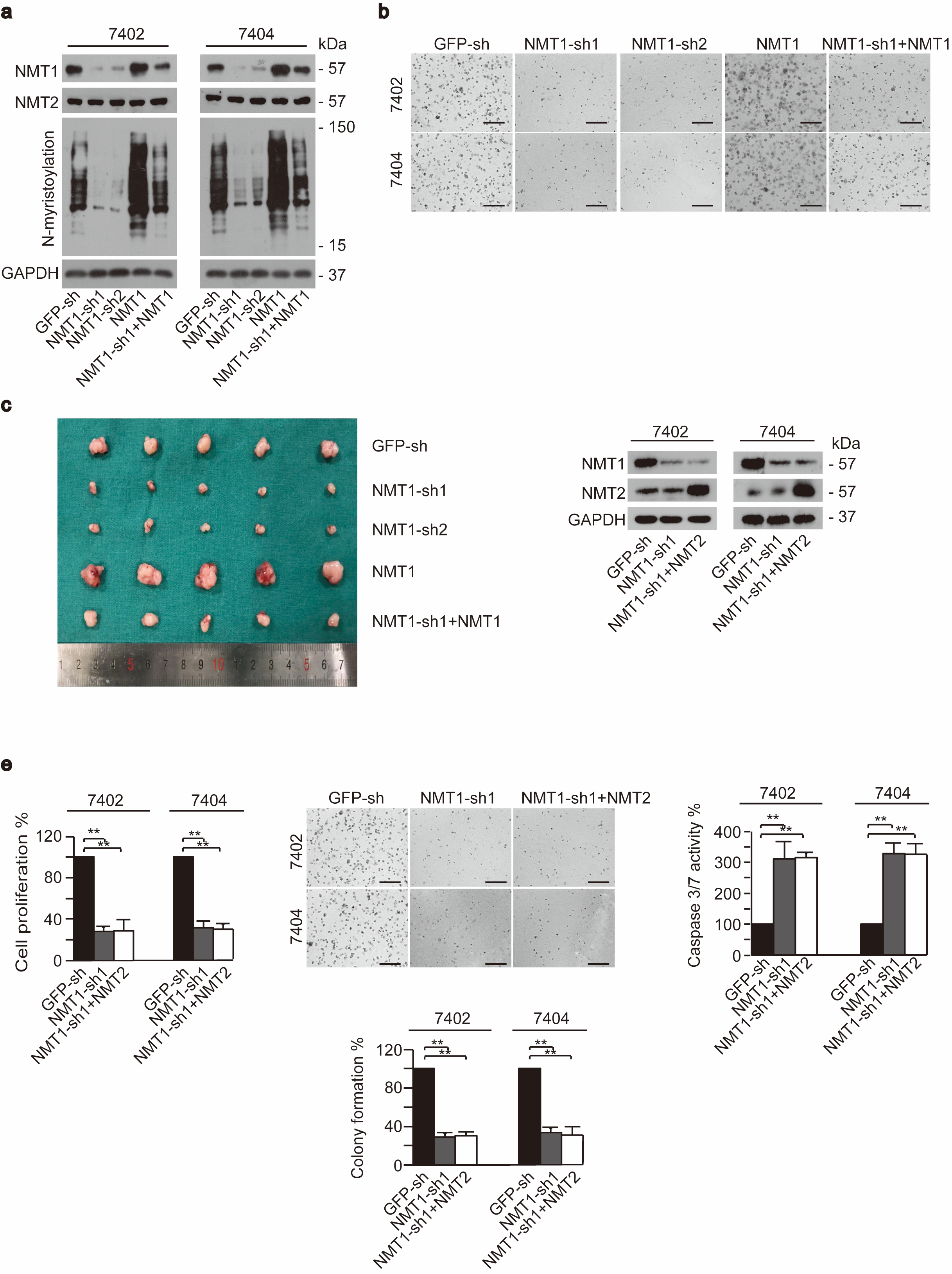


**Supplementary Figure. S1. NMT1 and N-myristoylation are critical for maintaining transformative phenotypes**

(A) Effects of NMT1 knockdown and overexpression on NMT1, NMT2 and N-myristoylation. Representative images of NMT1, NMT2 and global N-myristoylation in control cells, Bel-7402 and Bel-7404 cells with NMT1 knocked down or overexpressed, as indicated. The global N-myristoylation was evaluated by CuAAC, and protein expressions were evaluated by WB.

(B) Representative images of soft ager colony formation experiments in control cells, Bel-7402 and Bel-7404 cells with NMT1 knocked down or overexpressed, as indicated. Scale bar, 500μm.

(C) Image of xenograft generated by control cells, Bel-7404 cells with NMT1 knocked down or overexpressed, as indicated. The data was graphed in Figure. 1D. n=5/group.

(D) NMT1 had no effects on NMT2. Representative WB images of NMT1 and NMT2 in control cells, Bel-7402 and Bel-7404 cells with NMT1 knocked down or overexpressed, as indicated.

(E) NMT1-reduced transformative phenotypes could not be reversed by NMT2. Capacities of cell proliferation and colony formation, as well as Caspase 3/7 activity were measured by an MTT-based assay, soft agar colony formation assay, and Caspase 3/7 Glo reagent, respectively, in control cells, Bel-7402 and Bel-7404 cells with NMT1 knocked down with or without simultaneous overexpression of NMT2. Scale bar, 500μm.

The data are shown as the means + SD from 3 independent experiments (Supplementary Figure. S1E). Images of WB and soft agar are representative ones of 3 independent experiments. The data from Supplementary Figure. S1E were analyzed by a one-way ANOVA test. **, p < 0.01 indicate statistical significance.

**Supplementary Figure. S2.**


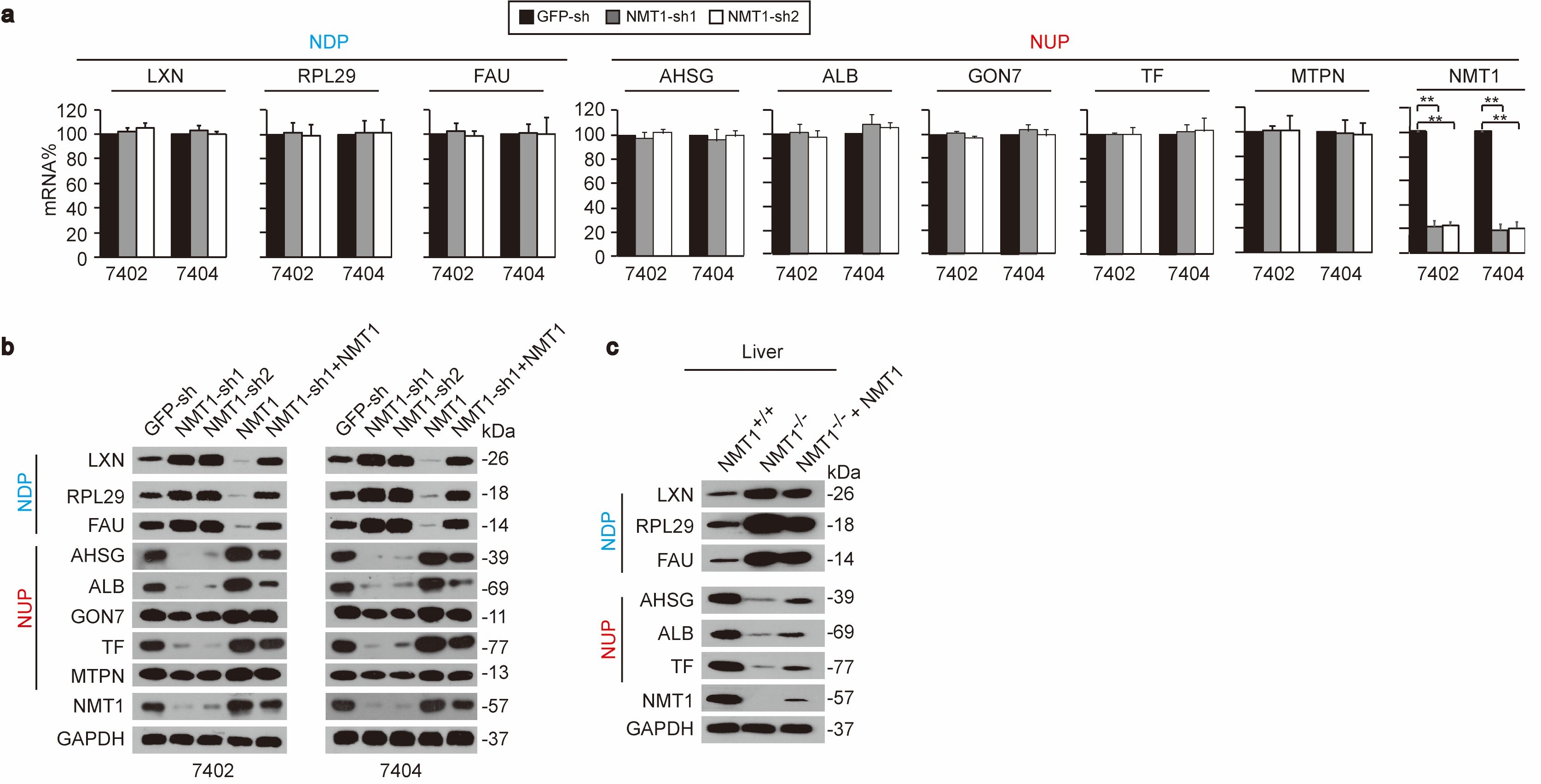


**Supplementary Figure. S2. NMT1 oppositely regulated NDP and NUP via an mRNA-independent manner**

(A) mRNAs of NDP and NUP in control cells, Bel-7402 and Bel-7404 cells with NMT1 knocked down by NMT1-sh1 and –sh2, respectively, as measured by qPCR.

(B) NMT1 oppositely controlled protein levels of NDP and NUP in liver cancer cells. Protein levels of NDP and NUP in control cells, Bel-7402 and Bel-7404 cells with NMT1 knocked down or overexpressed, as measured by WB.

(C) NMT1 negatively regulated NDP while positively regulated NUP in the liver of mouse. NDP and NUP were measured by WB in the liver of control mice, and mice with NMT1 knocked out with or without overexpressed exogenous NMT1.

The data are shown as the means + SD from 3 independent experiments (Supplementary Figure. S2A). Images of WB are representative ones of 3 independent experiments. The data from Supplementary Figure. S2A were analyzed by a one-way ANOVA test.

**Supplementary Figure. S3.**


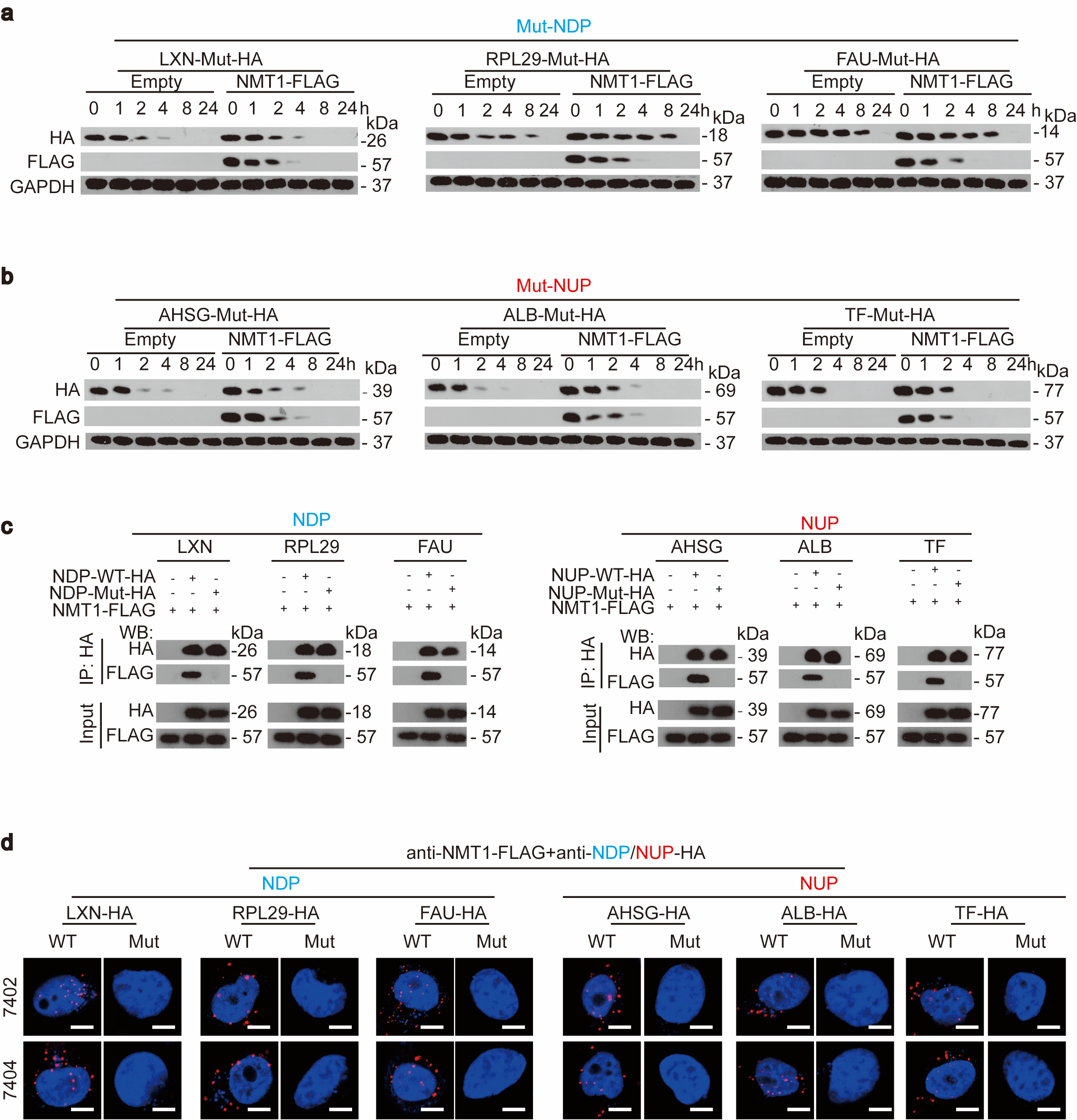


**Supplementary Figure. S3. NMT1 exerted its function via the NDP/NUP motifs.**

(A-B) Mutation of the NDP and NUP motifs blocked the effects by NMT1 on protein half-life. CHX chase experiments were performed in Bel-7402 cells expressing NDP/NUP without functional NDP/NUP motifs (Mut) under the treatment with or without overexpression of NMT1.

(C) The NDP/NUP motifs were critical for the interaction between NMT1 and NDP/NUP. Exogenous NDP/NUP-HA with (WT) or without functional NDP/NUP motifs (Mut) and exogenous NMT1-FLAG were co-expressed in Bel-7402 cells. NDP/NUP-HA were immuno-precipitated by anti-HA antibodies, and co-immuno-precipitation of NMT1-FLAG were evaluated by anti-FLAG antibodies.

(D) PLA revealed that the NDP/NUP motifs were essential for binding between NMT1 and NDP/NUP. Exogenous NDP/NUP-HA with (WT) or without functional NDP/NUP motifs (Mut) and exogenous NMT1-FLAG were co-expressed in Bel-7402 cells. The direct interactions between exogenous NDP/NUP and NMT1 were measured by PLA using anti-FLAG and anti-HA antibodies. Scale bar, 20μm.

Images of WB and PLA are representative ones of 3 independent experiments.

**Supplementary Figure. S4**


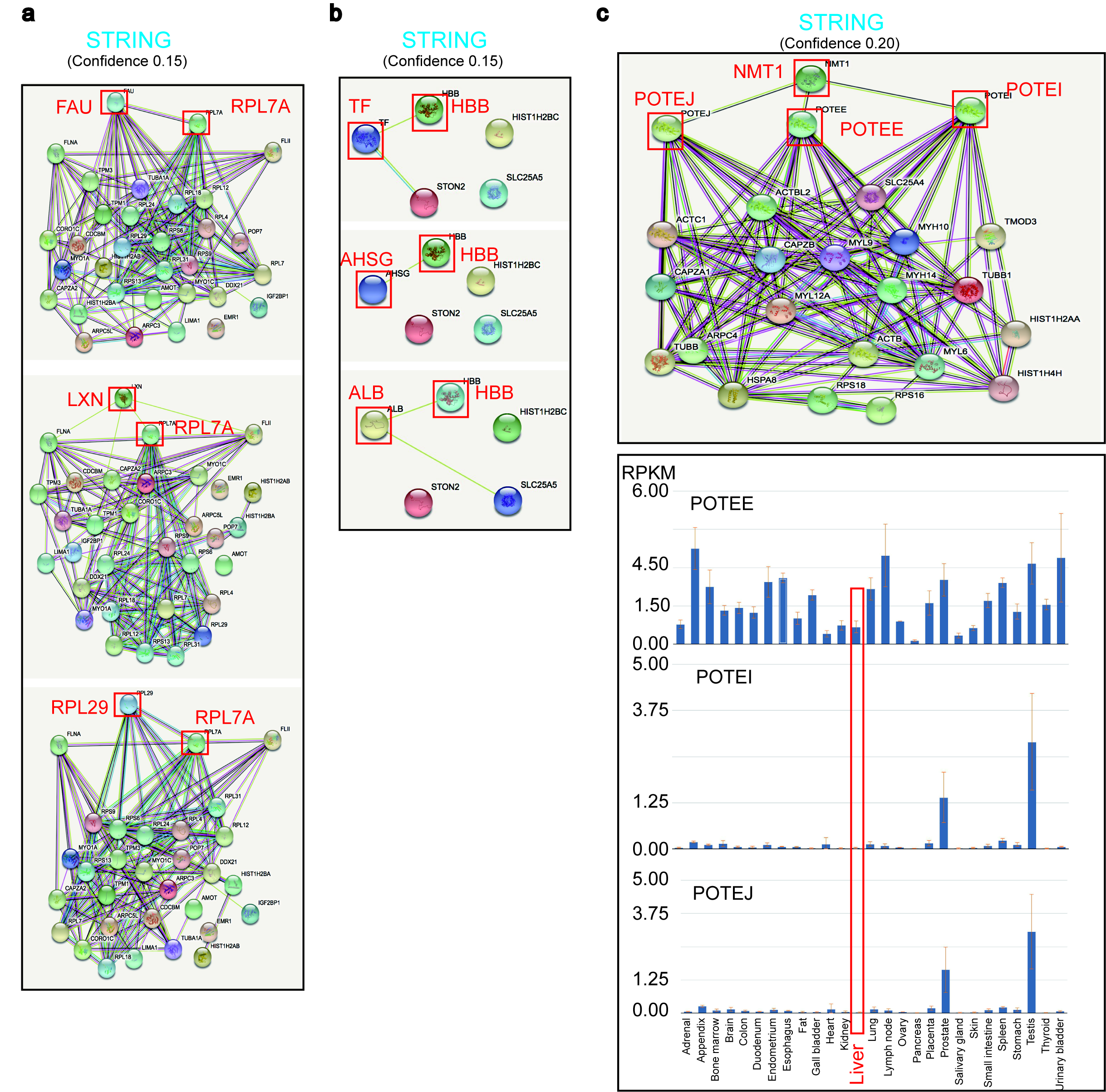


**Supplementary Figure. S4. RPL7A, HBB and POTE family were identified to interact with NDP and NUP**

(A) The potential interaction between RPL7A and NDP (including FAU, LXN and RPL29) was confirmed by STRING at a confidence of 0.15.

(B) The potential interaction between HBB and NUP (including TF, AHSG and ALB) was confirmed by STRING at a confidence of 0.15.

(C) The interaction between NMT1 and POTE family was identified by STRING at a confidence of 0.20 (upper). The expression pattern of POTE family (including POTEE, POTEI and POTEJ) in indicated organs was revealed using the public available data (BioProject: PRJEB4337) from Fagerberg L et al [Ref.1].

**Supplementary Figure. S5**


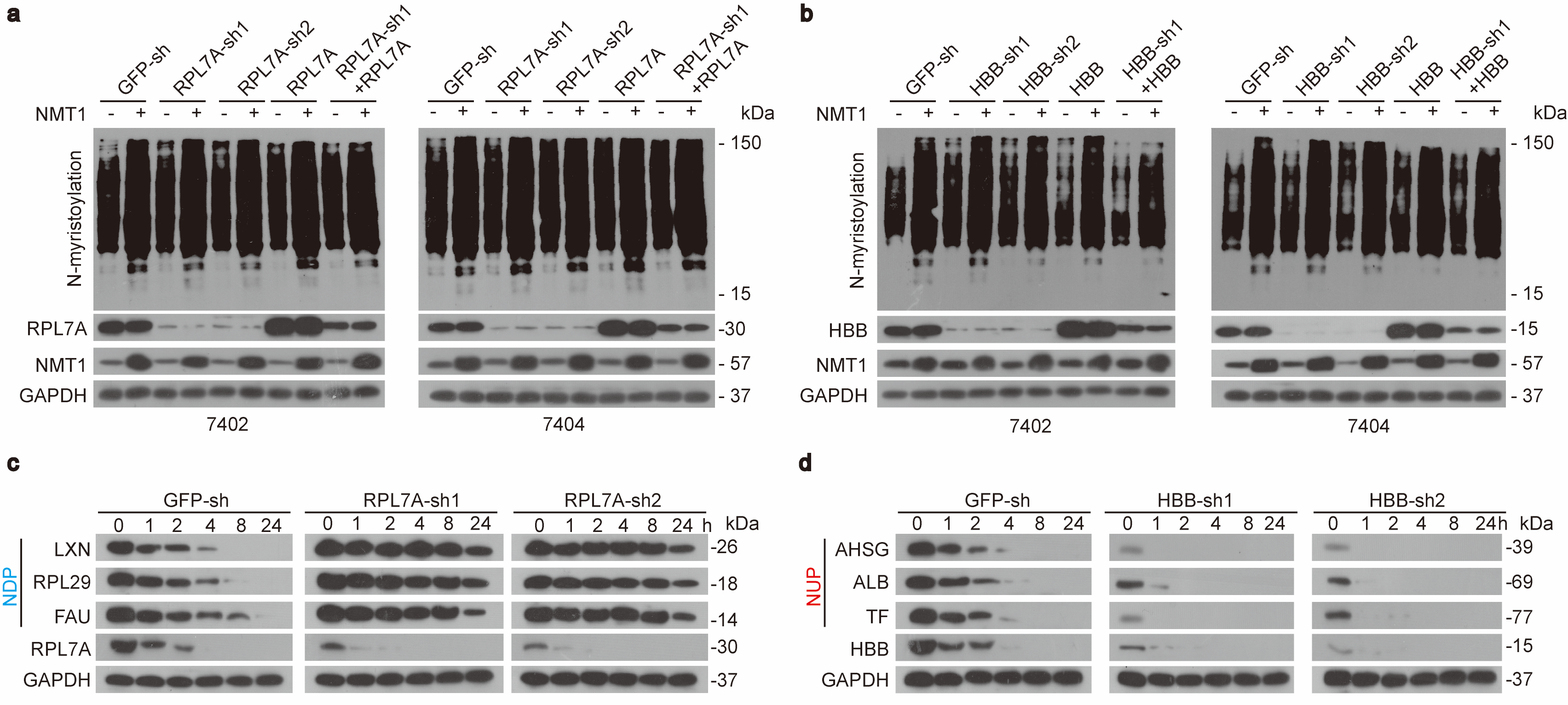


**Supplementary Figure. S5. The regulation of RPL7A and HBB on NDP and NUP**

(A) RPL7A didn’t control global N-myristoylation. Global N-myristoylation was evaluated by WB and CuAAC in control cells, Bel-7402 and Bel-7404 cell with RPL7A knocked down or overexpressed, with or without overexpression of NMT1.

(B) HBB didn’t control global N-myristoylation. The global N-myristoylation was evaluated by WB and CuAAC in control cells, Bel-7402 and Bel-7404 cell with HBB knocked down or overexpressed, with or without overexpression of NMT1.

(C) Knocking RPL7A down prolonged half-life of NDP. The CHX-chase experiments were performed in control cells and Bel-7402 cells with RPL7A knocked down. The expression of NDP was evaluated by WB.

(D) Knocking HBB down shortened half-life of NUP. The CHX-chase experiments were performed in control cells and Bel-7402 cells with HBB knocked down. The expression of NUP was evaluated by WB.

Images of WB are representative ones of 3 independent experiments.

**Supplementary Figure. S6**


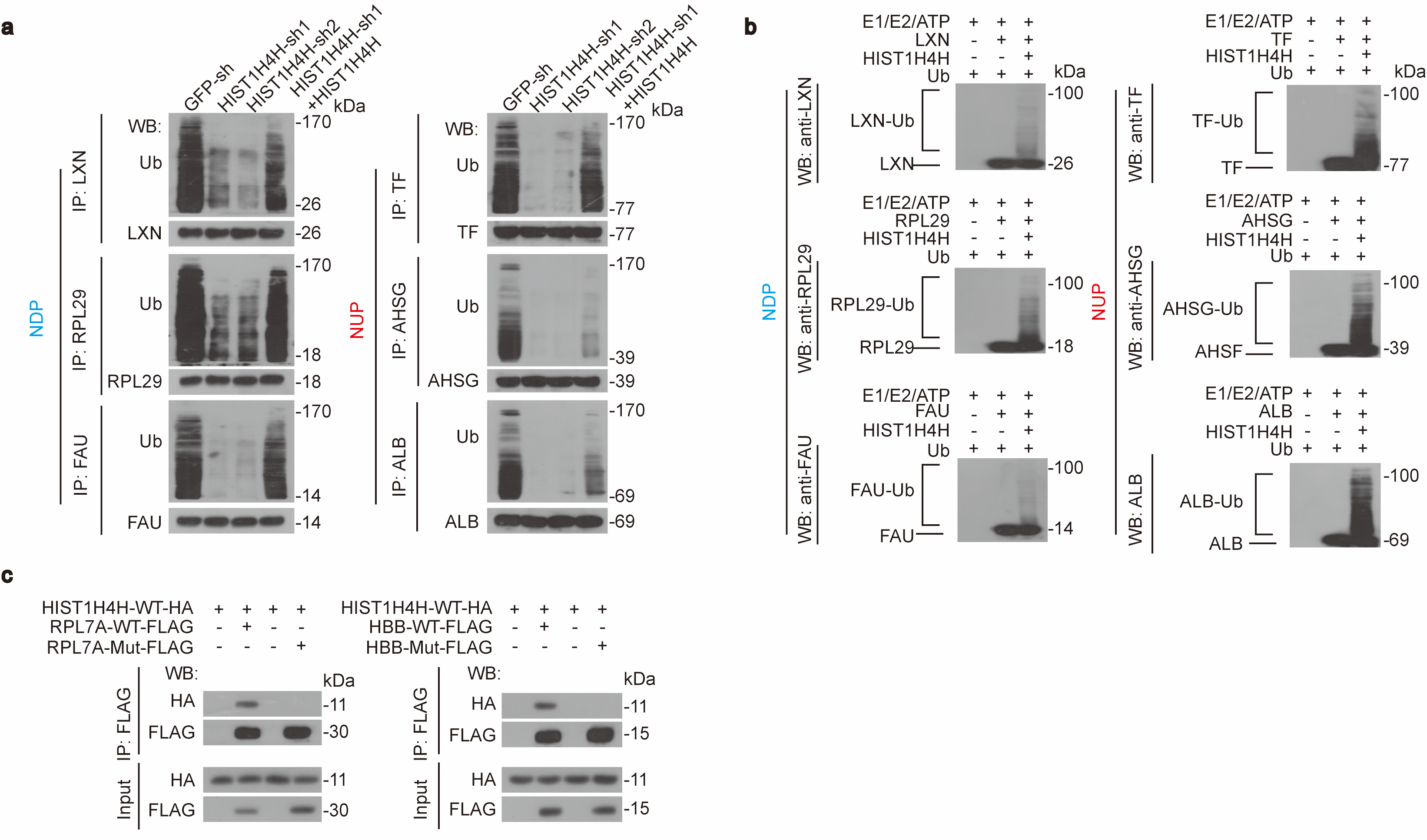


**Supplementary Figure. S6. HIST1H4H is critical for ubiquitination for NDP and NUP**

(A) *In vivo* ubiquitination assay demonstrated that HIST1H4H stimulates ubiquitination of both NDP and NUP. Ubiquitination of NDP and NUP was measured in control cells and Bel-7402 cells with HIST1H4H knocked down in the presence or absence of simultaneous overexpression of HIST1H4H, by firstly immuno-precipitation of NDP/NUP by corresponding antibodies, as indicated, followed by WB using anti-Ub antibodies.

(B) *In vitro* ubiquitination assay demonstrated that NDP and NUP could be ubiquitinated by HIST1H4H. Purified E1/E2 (UBE2S) /ATP, NDP or NUP, Ub with or without purified HIST1H4H were incubated together at 37°C for 30min. The ubiquitination of purified NDP/NUP were measured by WB using corresponding antibodies, as indicated.

(C) RPL7A and HBB interacted with HIST1H4H. WT or mutant RPL7A/HBB (without functional PPVxxAxxxxV motif), as indicated, was co-expressed with WT HIST1H4H in Bel-7402 cells. The exogenous RPL7A/HBB-FLAG was immuno-precipitated by anti-FLAG antibodies, and co-immuno-precipitation of exogenous HIST1H4H-HA was visualized by WB using anti-HA antibodies.

Images of WB are representative ones of 3 independent experiments.

**Supplementary Figure. S7**


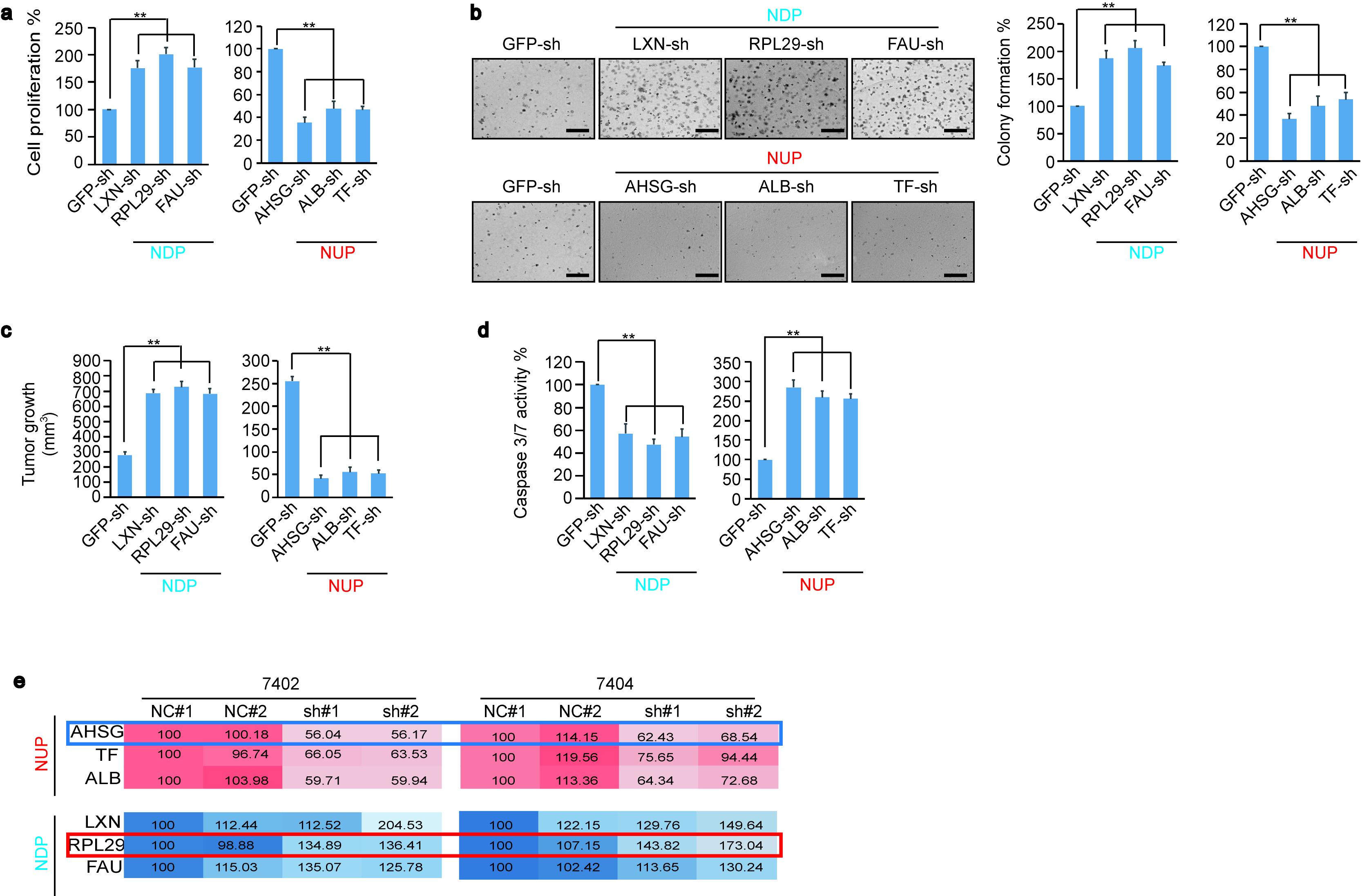


**Supplementary Figure. S7. NDP and NUP play opposite roles in maintaining transformative phenotypes in liver cancer cells**

(A-D) Transformative phenotypes of liver cancer cells, including capacities of cell proliferation (A), colony formation (B), *in vivo* xenograft growth (C), and Caspase 3/7 activity (D) were measured by an MTT-based assay (A), soft agar colony formation assay (B), xenograft mouse model (C) and Caspase 3/7 Glo luciferase reagent (D), respectively, in Bel-7402 cells.

(E) Relative expression of NUP (AHSG, TF and ALB) and NDP (LXN, RPL29 and FAU) in control cells [treated with negative control shRNA (NC)] and Bel-7402 cells with NMT1 knocked down [treated by NMT1-shRNA (sh)]. The data were from iTraq (two biological replicates).

The data are shown as the means + SD from 3 independent experiments (Supplementary Figure. S7A-D). Images of soft agar are representative ones of 3 independent experiments. **, p < 0.01 indicate statistical significance. The data from Supplementary Figure. S7A-D were analyzed by a one-way ANOVA test.

**References**

1. Fagerberg L, et al. Analysis of the human tissue-specific expression by genome-wide integration of transcriptomics and antibody-based proteomics. Mol Cell Proteomics. 2014; 13: 397-406.
